# Supplementary figures and images for: Cuproptosis related ceRNA axis AC008083.2/miR-142-3p promotes the malignant progression of nasopharyngeal carcinoma through STRN3
Source: PeerJ. 2024 Aug 12;12:e17859. doi: 10.7717/peerj.17859 (PMC11326429; doi:10.7717/peerj.17859)

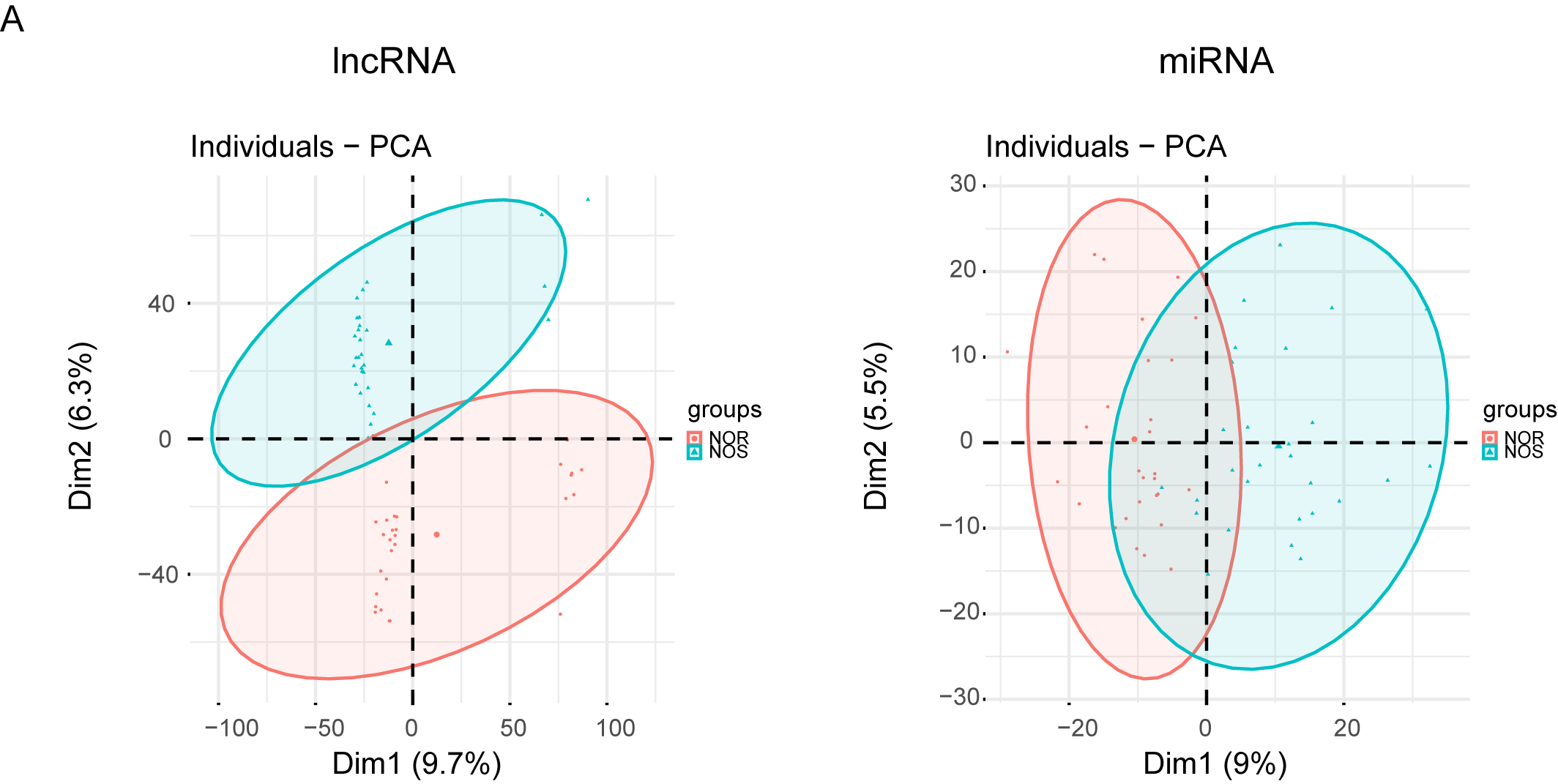

Supplement: Figure S1 [file peerj-12-17859-s001.png]

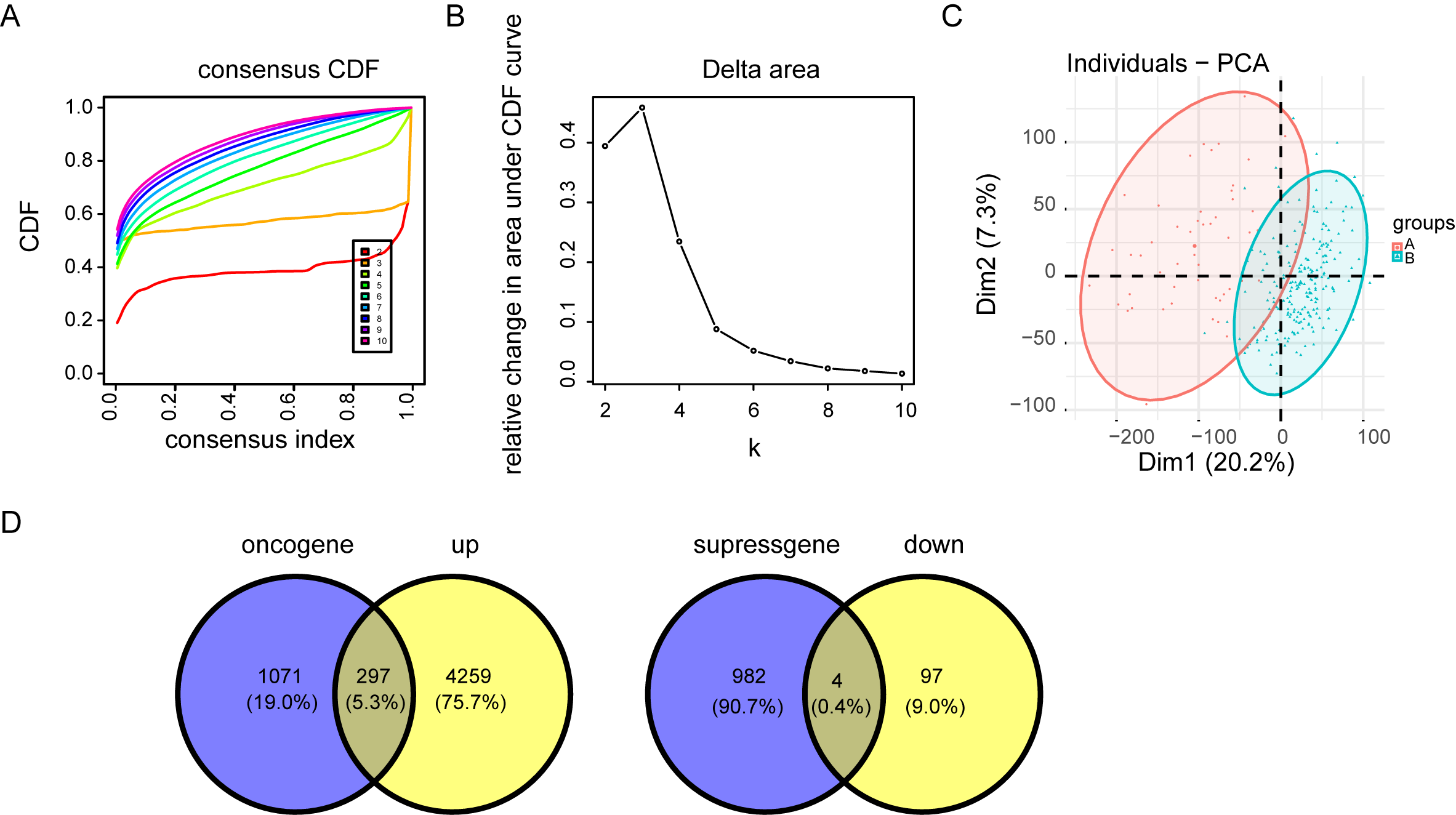

Supplement: Figure S2 — (A) Cumulative distribution function (CDF).(B) Delta area plot.(C) PCA depicted the distribution for clusters.(D) Venn diagrams displaying the intersection genes of the COX analysis and the DEG analysis of mRNAs [file peerj-12-17859-s002.png]

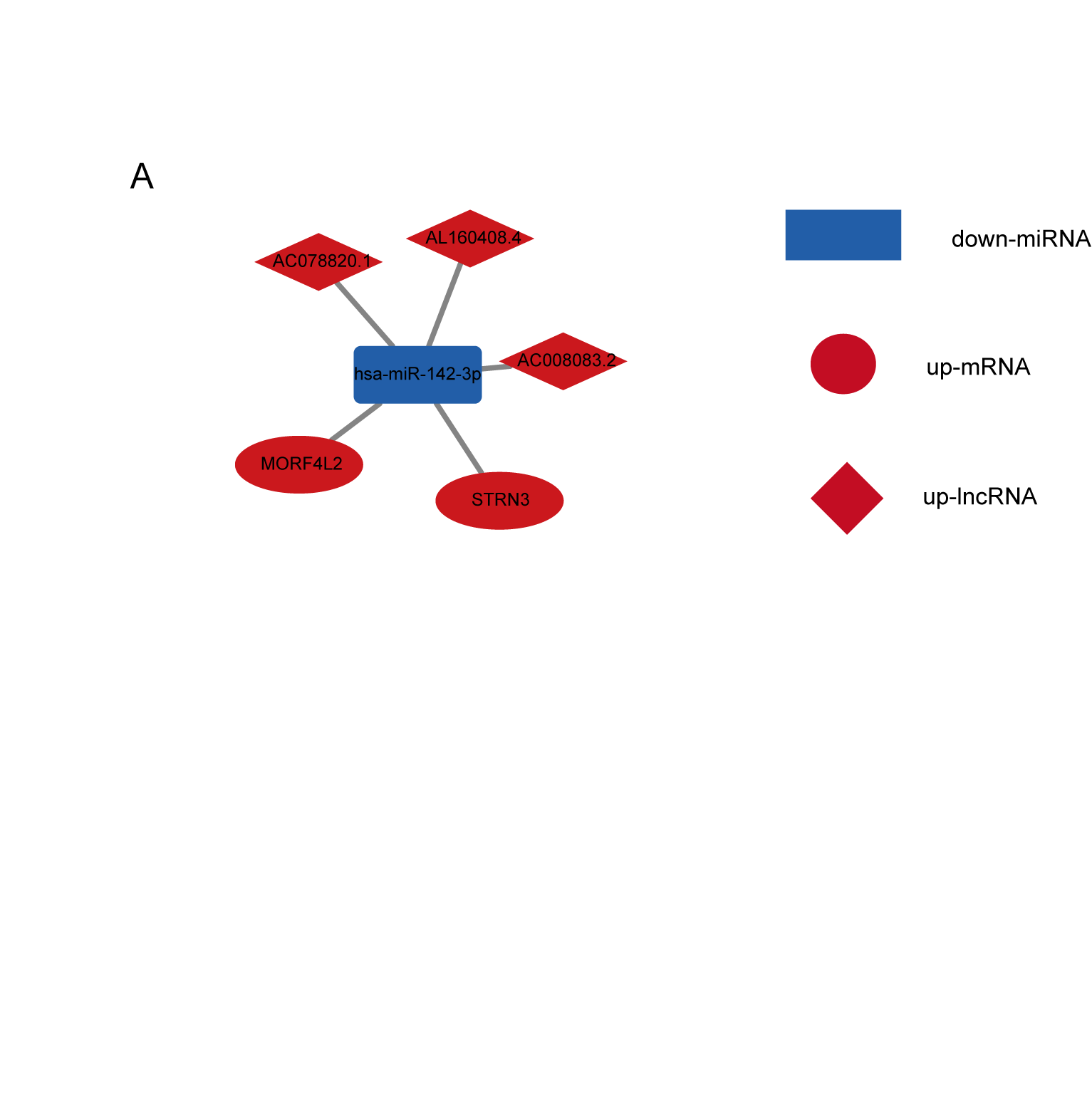

Supplement: Figure S3 [file peerj-12-17859-s003.png]

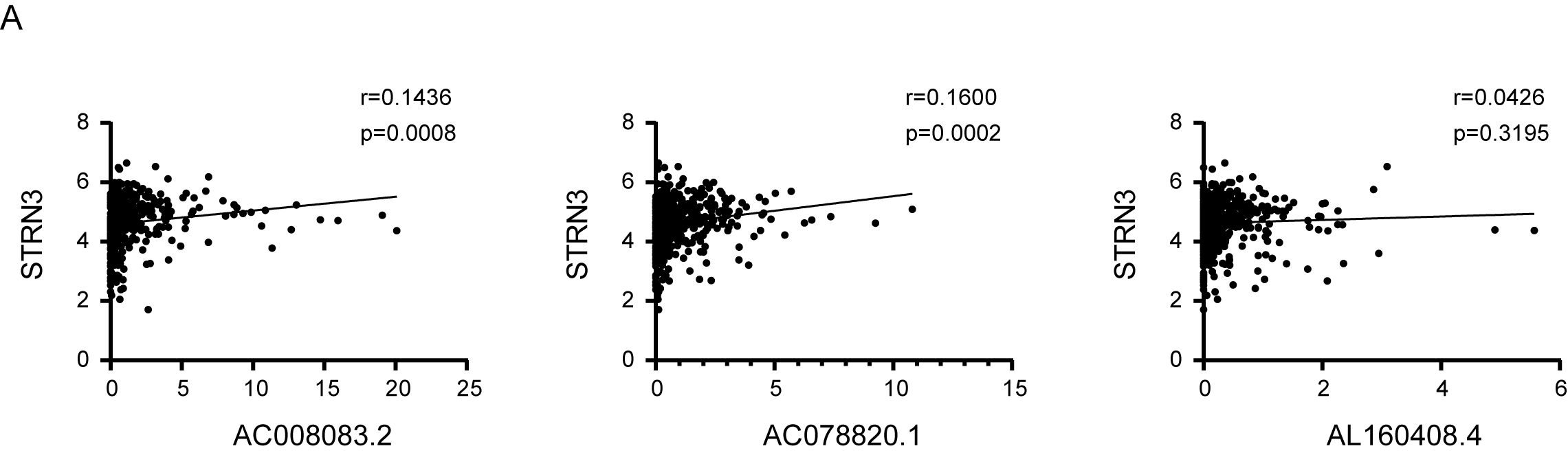

Supplement: Figure S4 [file peerj-12-17859-s004.png]

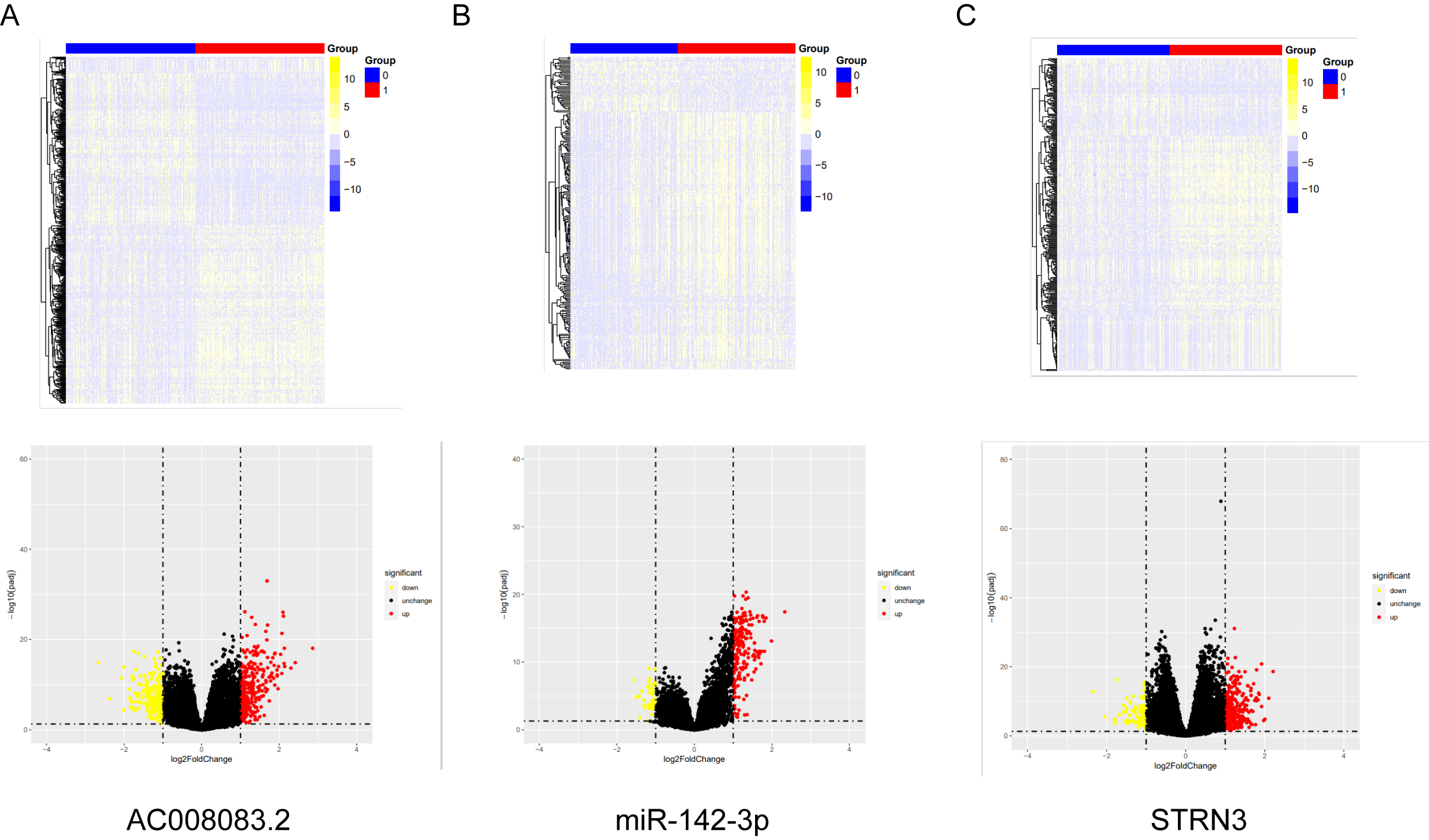

Supplement: Figure S5 — (A) The volcano map and heat map showed the differential genes in the lncRNA AC008083.2-high expression group and AC008083.2-low expression group. (B) The volcano map and heat map showed the differential genes in the miR-142-3p-high expression group and miR-142-3p-low expression group.(C)The volcano map and heat map showed the differential genes in the STRN3-high expression group and STRN3-low expression group. [file peerj-12-17859-s005.png]

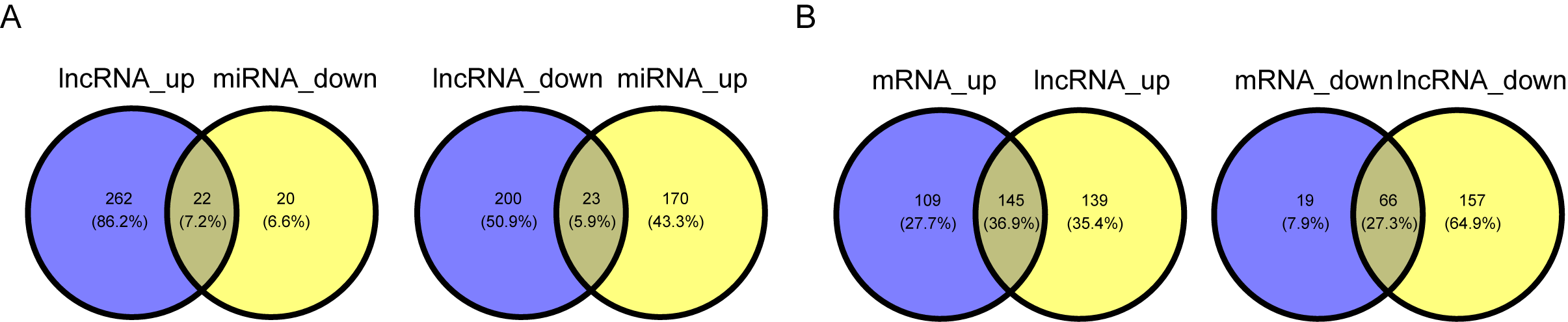

Supplement: Figure S6 — (A) Venn diagrams showed the overlapping genes of AC008083.2 groups and miR-142-3p groups.(B) Venn diagrams showed the overlapping genes of AC008083.2 groups and STRN3 groups. [file peerj-12-17859-s006.png]

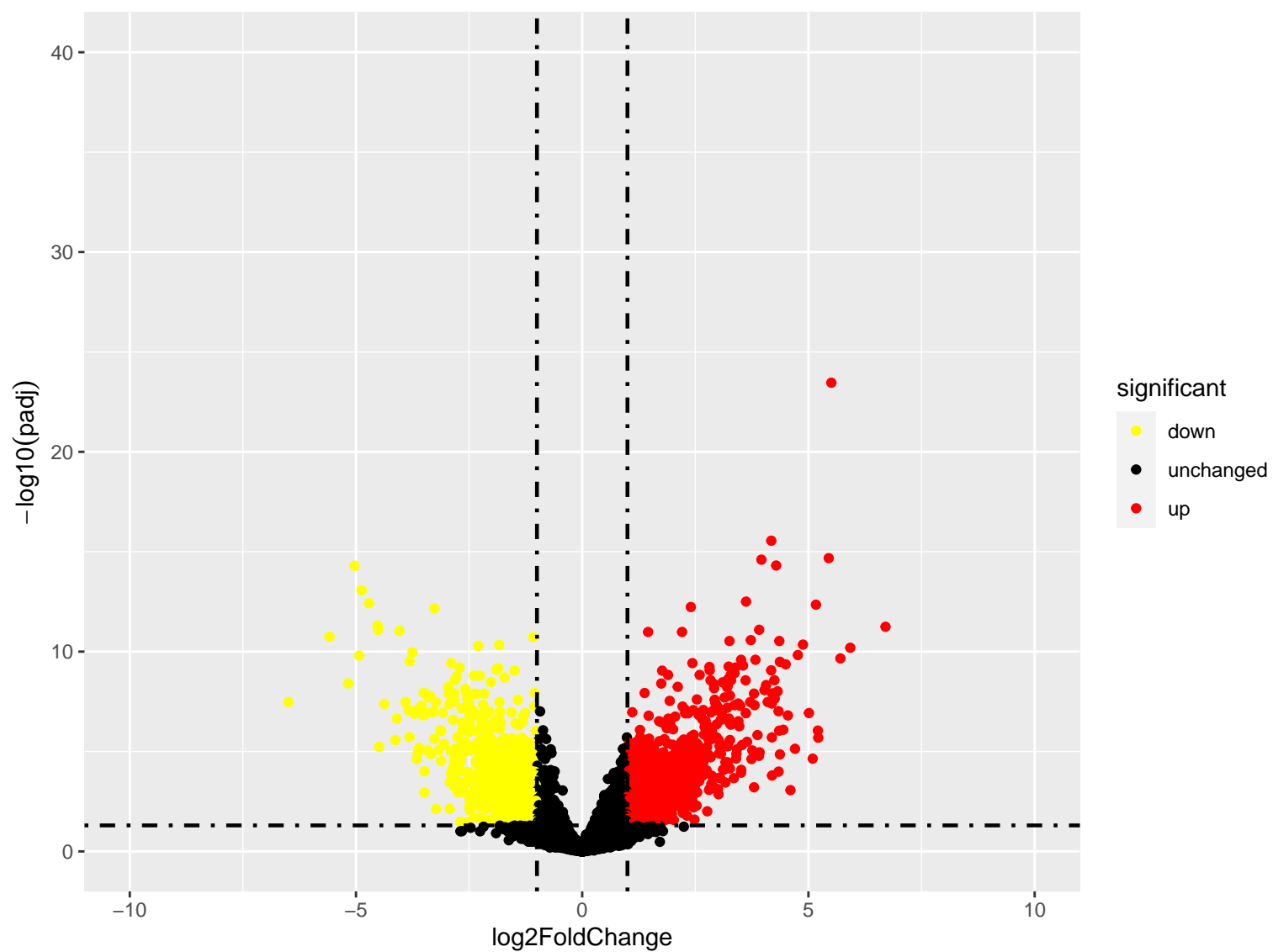

Supplement: Data S1 [file peerj-12-17859-s007.zip › Figure1/A1 lncrna_volcano-new.pdf]

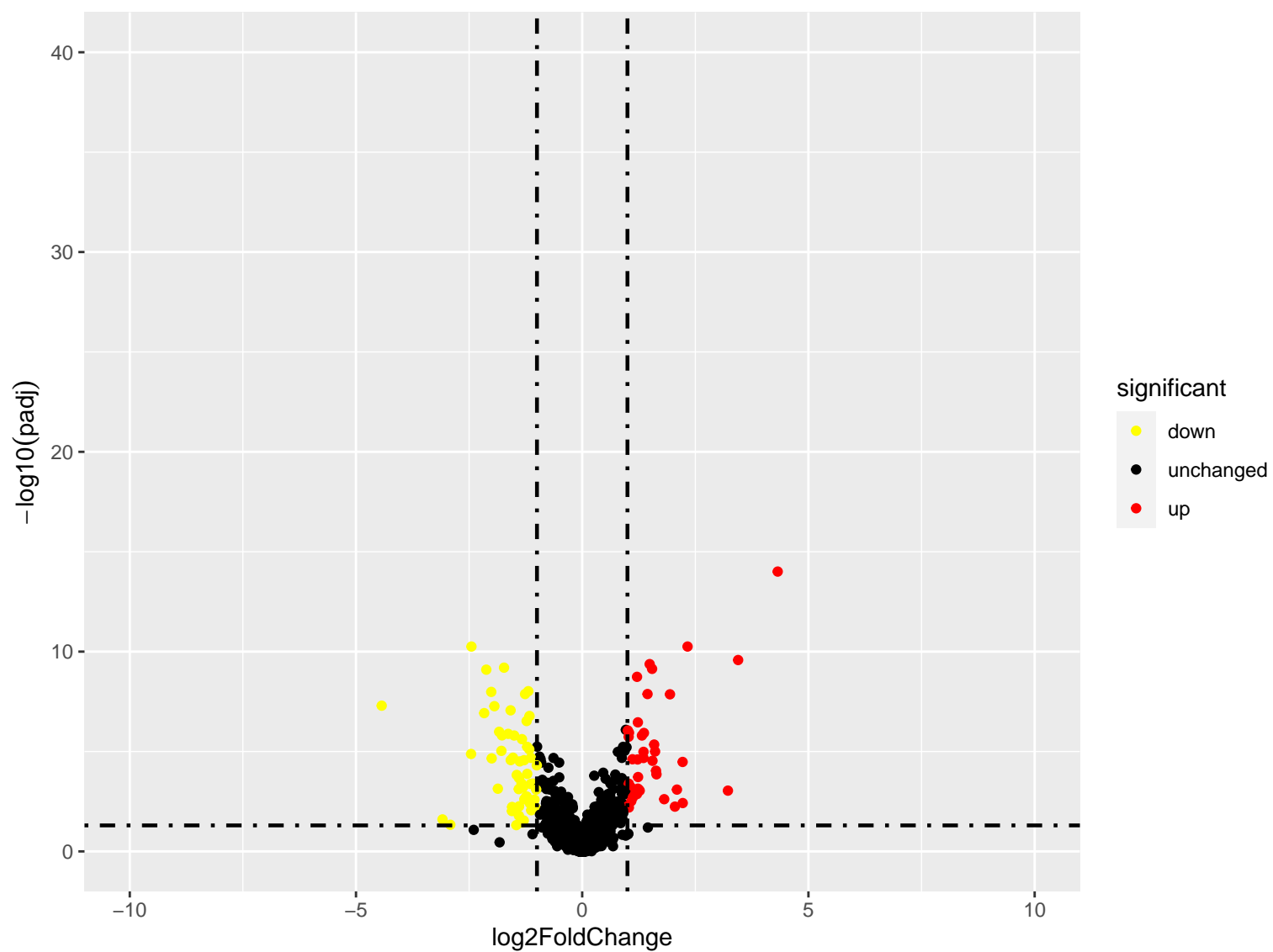

Supplement: Data S1 [file peerj-12-17859-s007.zip › Figure1/A2 nos2mi_volcano-new.pdf]

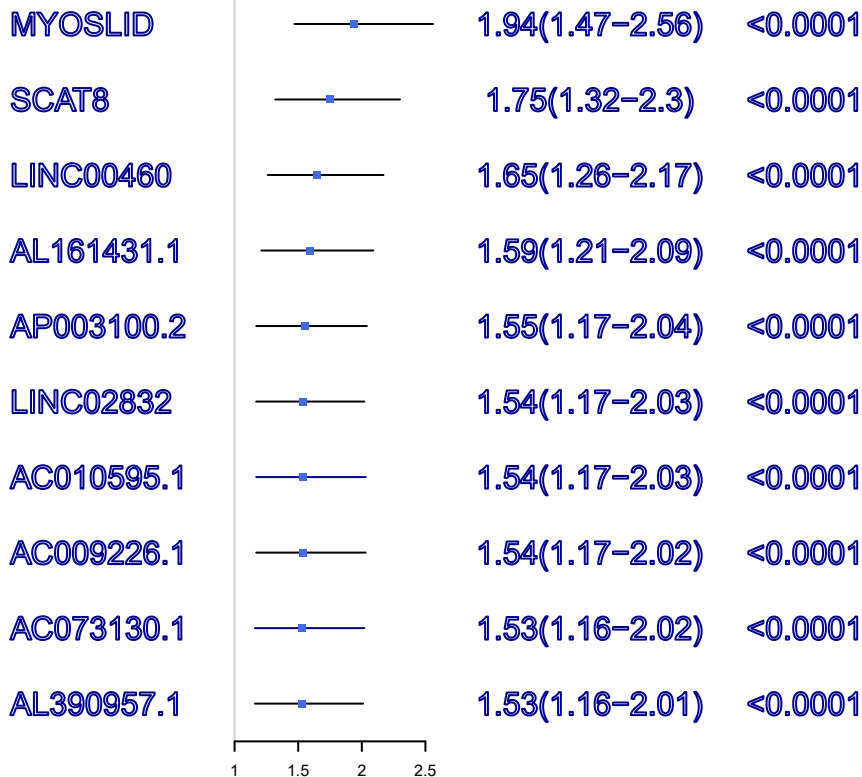

Supplement: Data S1 [file peerj-12-17859-s007.zip › Figure1/B1 Rplot-slt-new.pdf]

hsa-miR-30e-5p

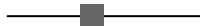

0.75(0.58-0.97)

0.03

hsa-miR-99a-3p

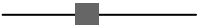

0.74(0.57-0.96)

0.02

hsa-let-7c-3p

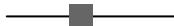

0.67(0.52-0.86)

<0.0001

0.525 0.6 0.675 0.75 0.825 0.9 0.975

Supplement: Data S1 [file peerj-12-17859-s007.zip › Figure1/B2 Rplot-slt-nos2.pdf]

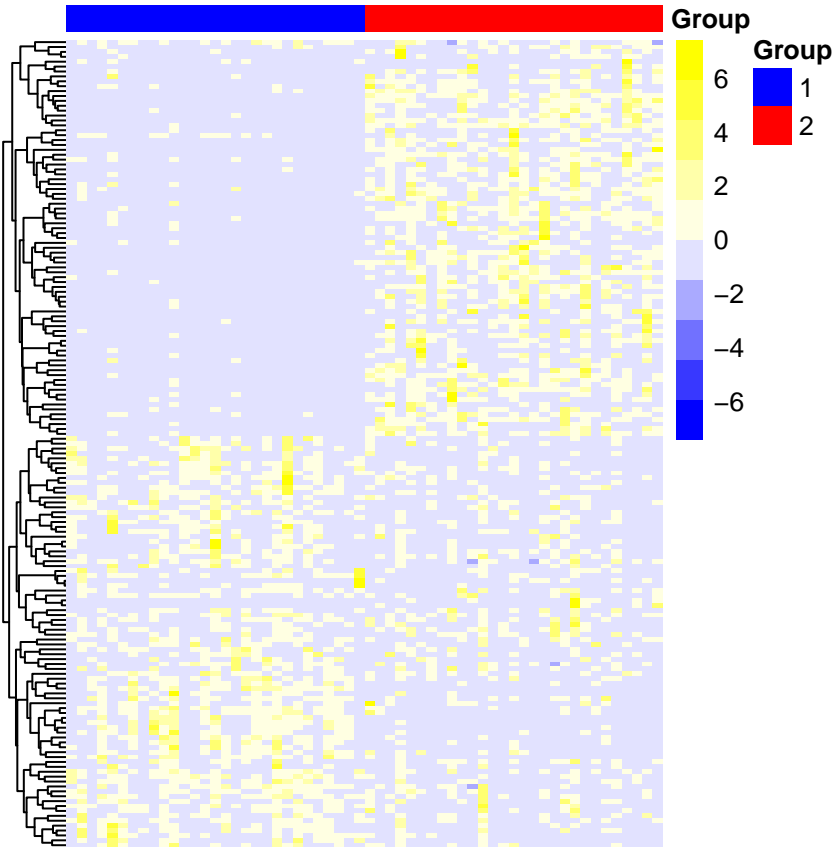

Supplement: Data S1 [file peerj-12-17859-s007.zip › Figure1/C1nos2-rt.pdf]

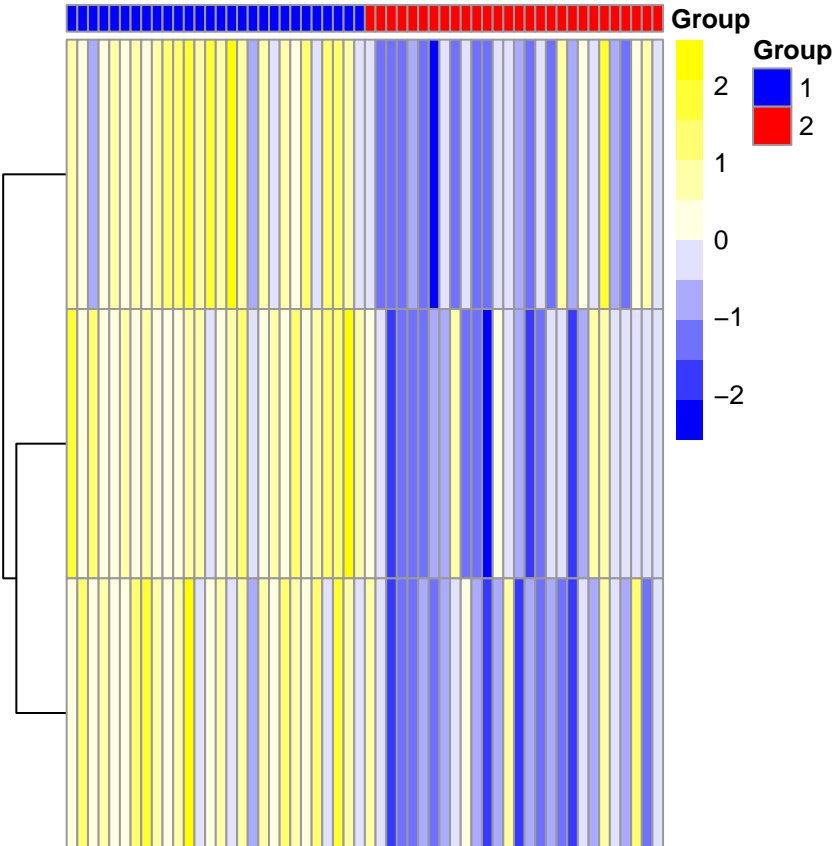

Supplement: Data S1 [file peerj-12-17859-s007.zip › Figure1/C2 nos2-rt.pdf]

consensus matrix k=3

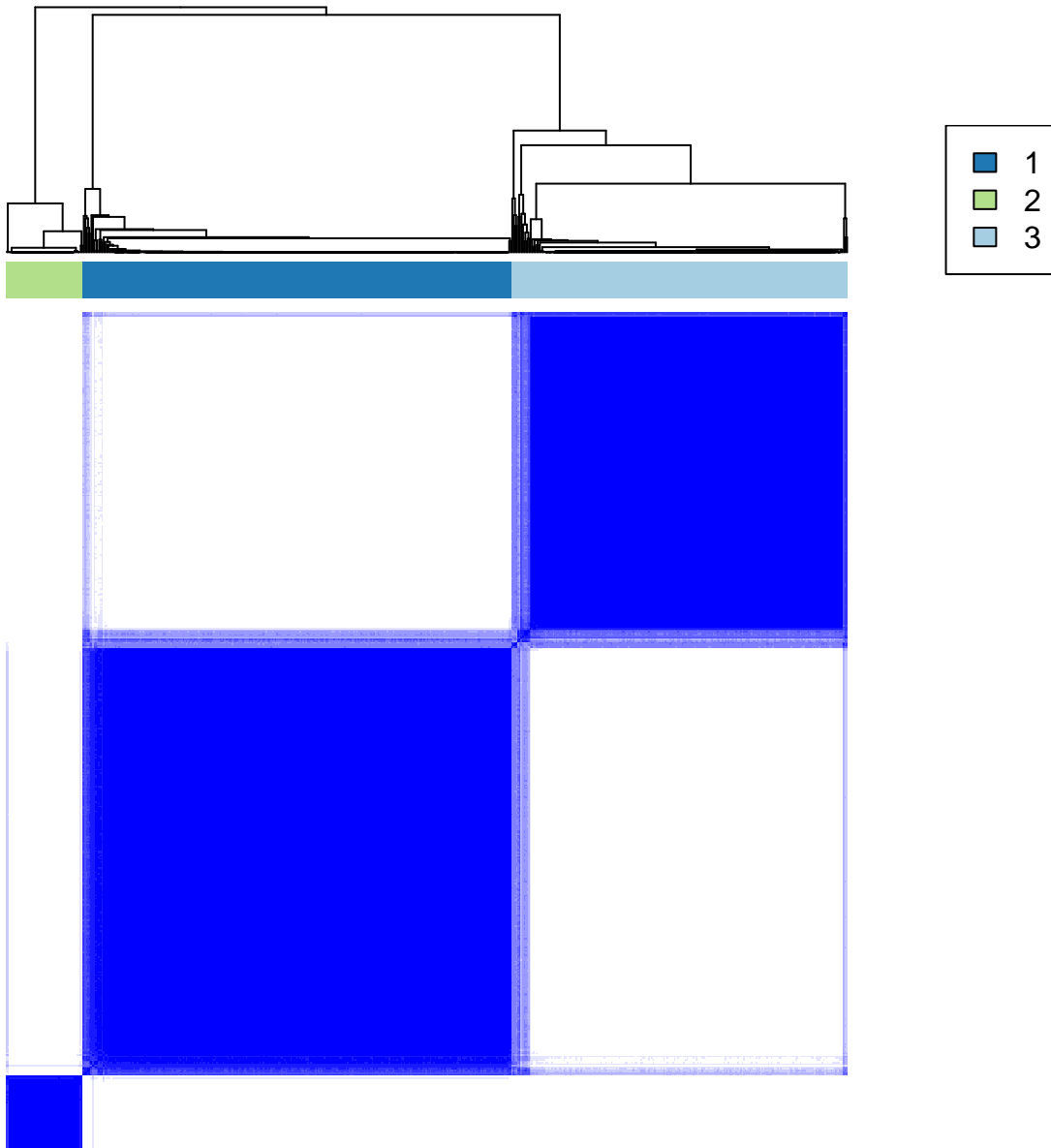

Supplement: Data S1 [file peerj-12-17859-s007.zip › Figure2/A consensus_3.pdf]

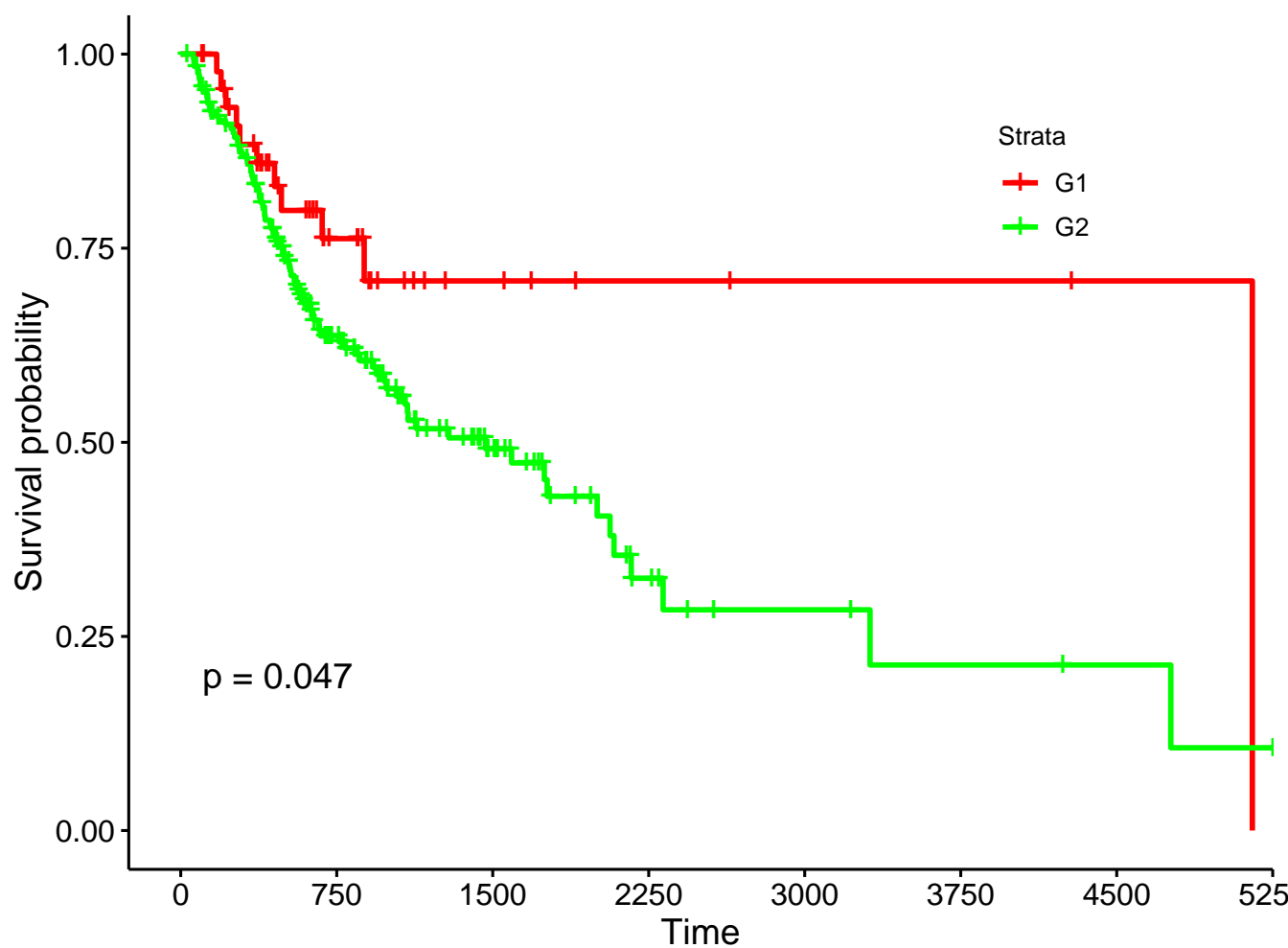

| Number at risk |    | 0   | 750 | 1500 | 2250 | 3000 | 3750 | 4500 | 5250 |
|----------------|----|-----|-----|------|------|------|------|------|------|
| Strata         | G1 | 46  | 18  | 6    | 3    | 2    | 2    | 1    | 0    |
|                | G2 | 191 | 86  | 33   | 10   | 5    | 3    | 2    | 1    |

Time

Supplement: Data S1 [file peerj-12-17859-s007.zip › Figure2/B Rplot.pdf]

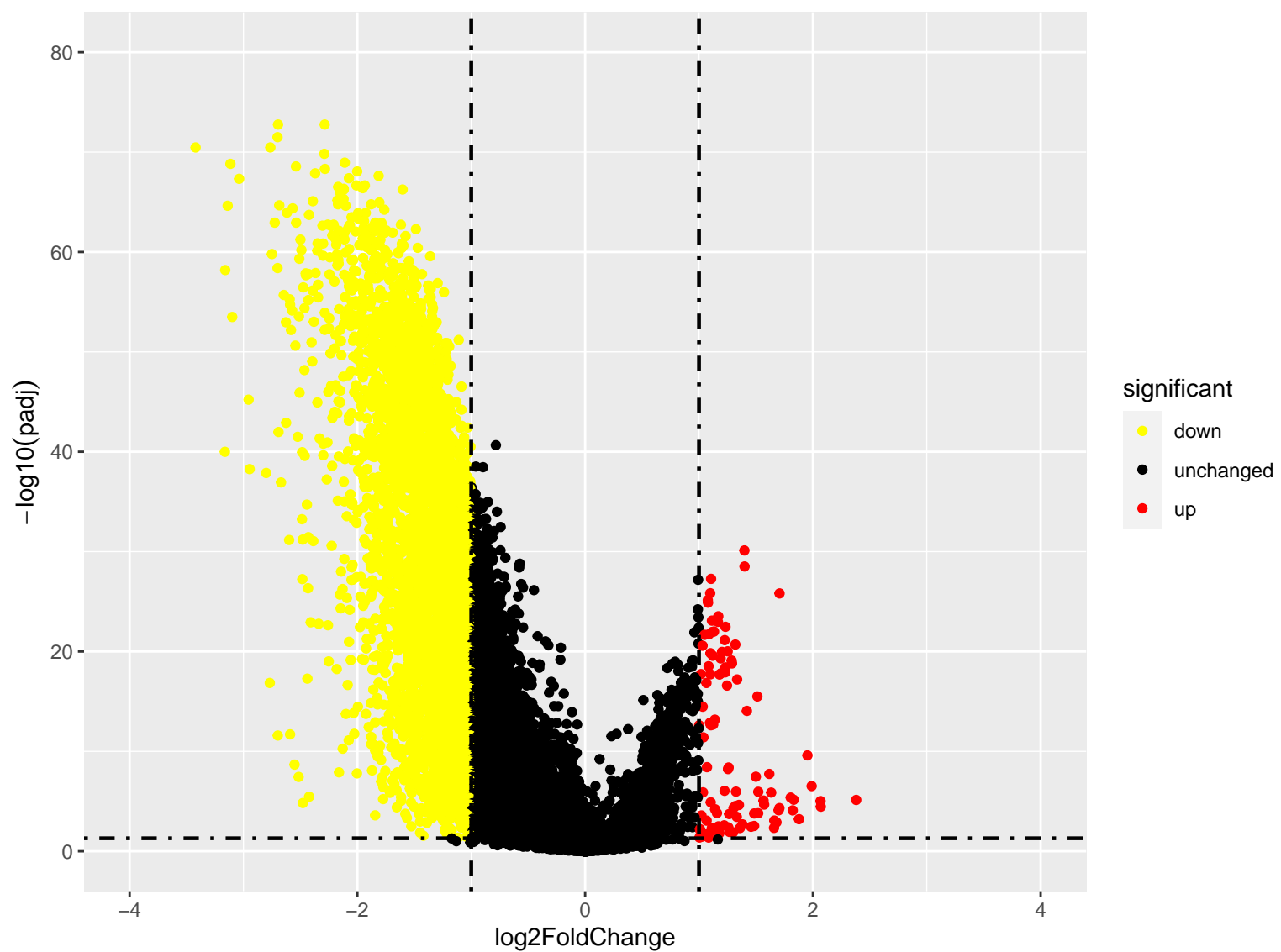

Supplement: Data S1 [file peerj-12-17859-s007.zip › Figure2/C Rplot01-hst-yellow.pdf]

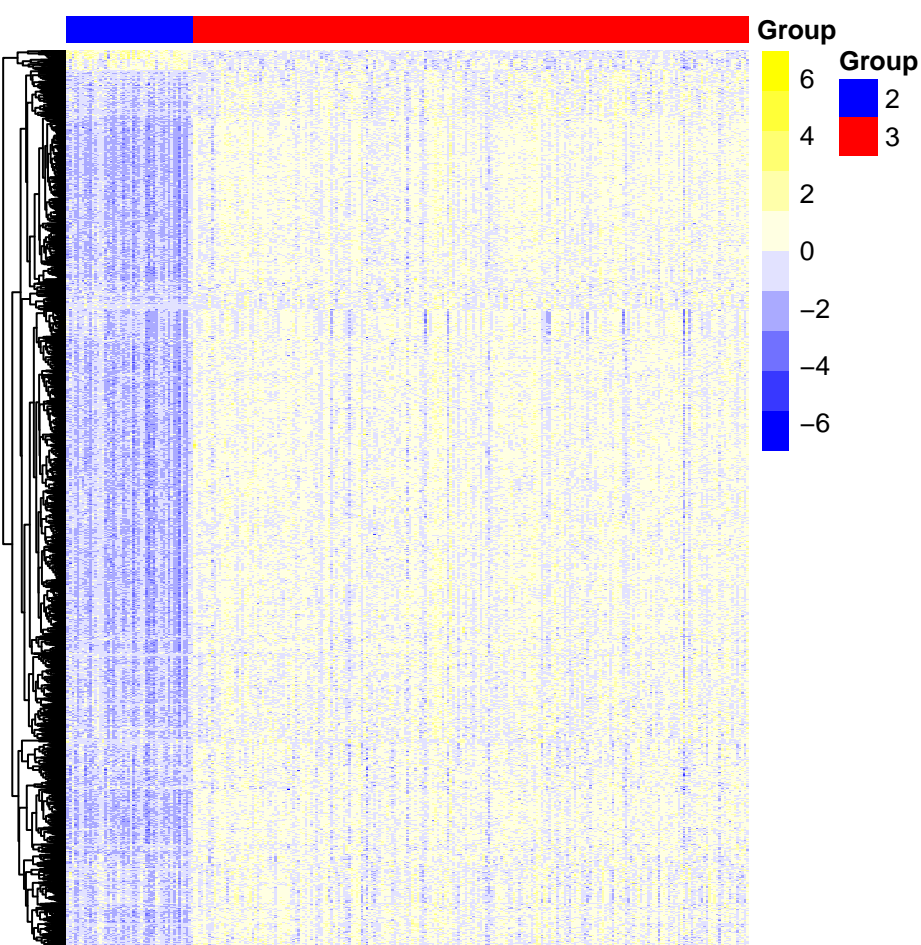

Supplement: Data S1 [file peerj-12-17859-s007.zip › Figure2/D Rplot01-new-yellow.pdf]

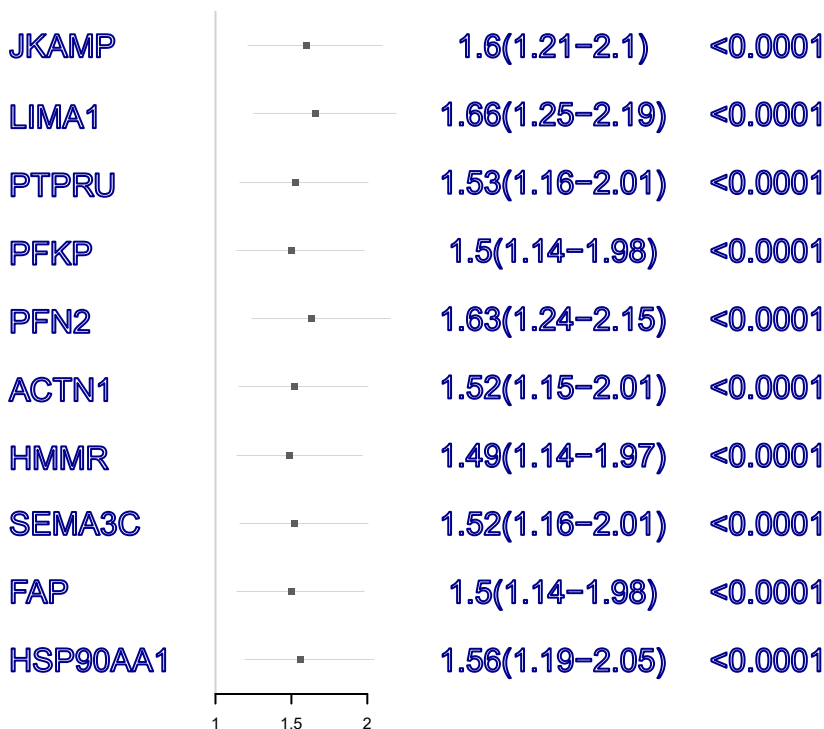

Supplement: Data S1 [file peerj-12-17859-s007.zip › Figure2/E Rplot-slt-m-overlap-1.pdf]

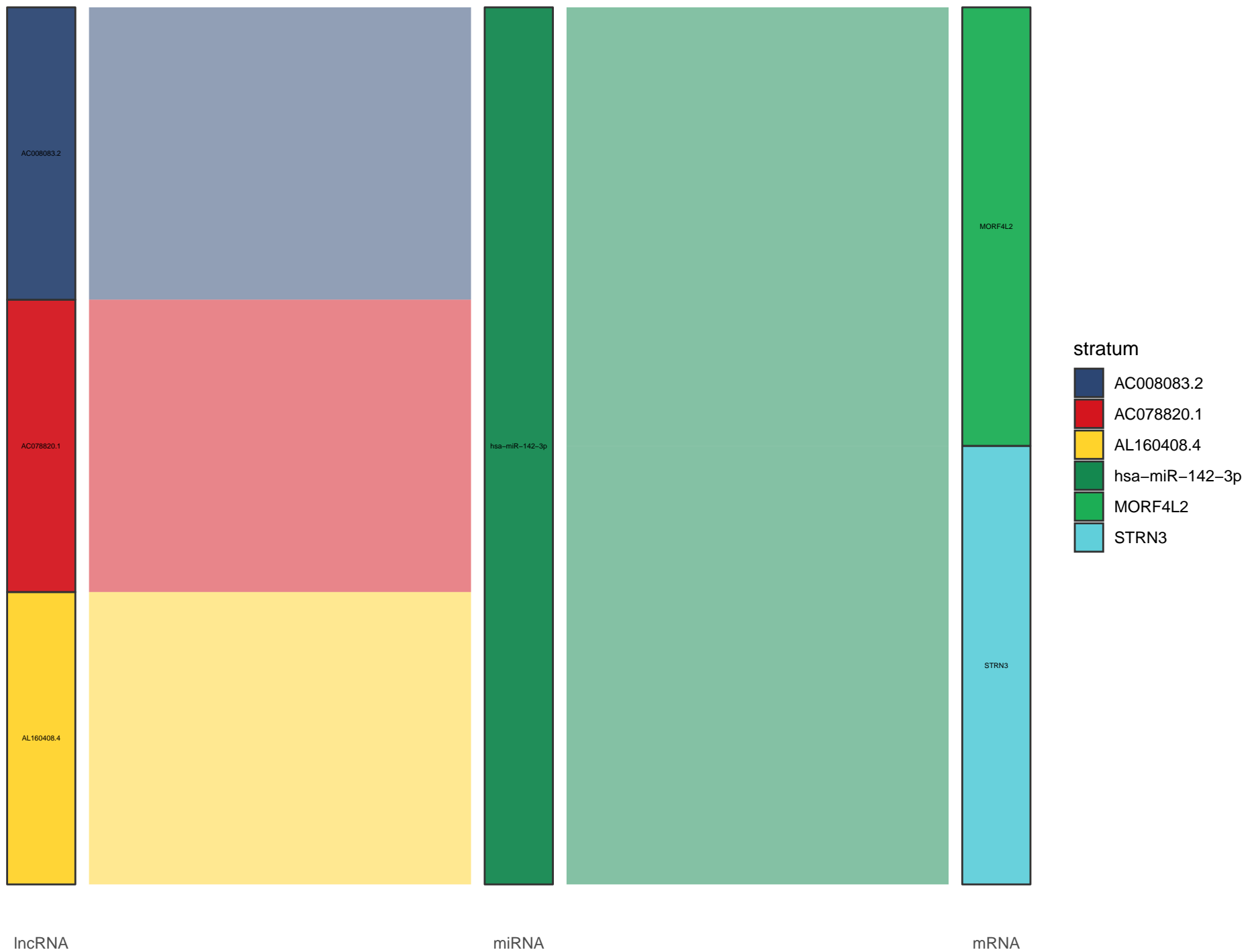

Supplement: Data S1 [file peerj-12-17859-s007.zip › Figure3/A Rplot02.pdf]

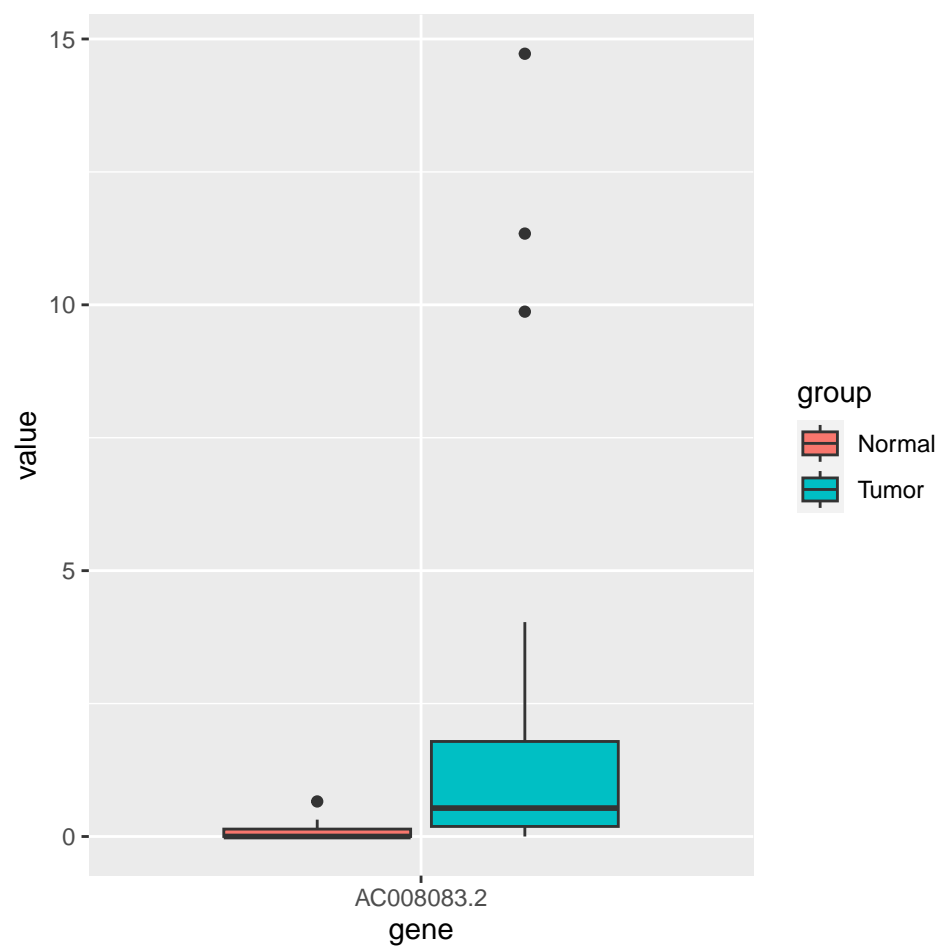

Supplement: Data S1 [file peerj-12-17859-s007.zip › Figure4/A/A1 AC008083.2.pdf]

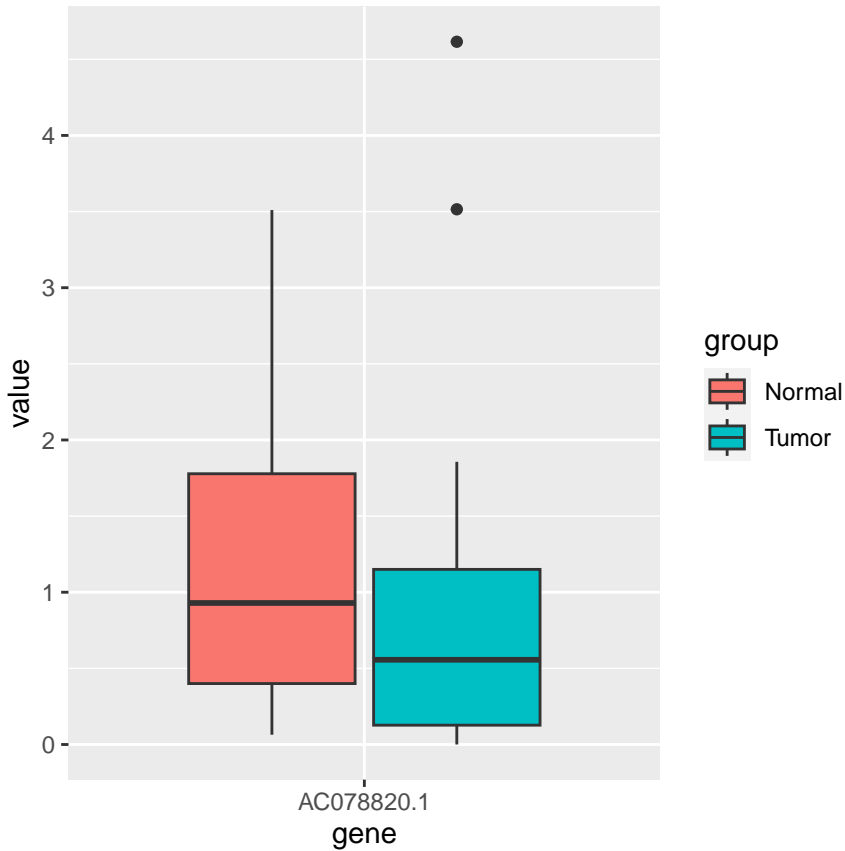

Supplement: Data S1 [file peerj-12-17859-s007.zip › Figure4/A/A2 AC078820.1.pdf]

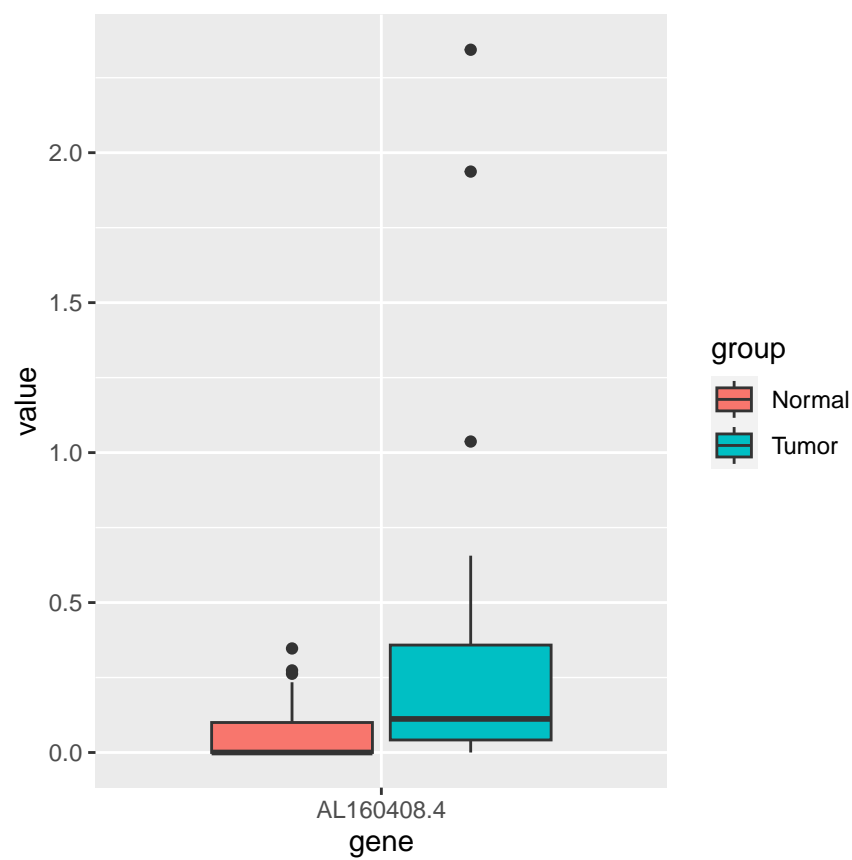

Supplement: Data S1 [file peerj-12-17859-s007.zip › Figure4/A/A3 AL160408.4.pdf]

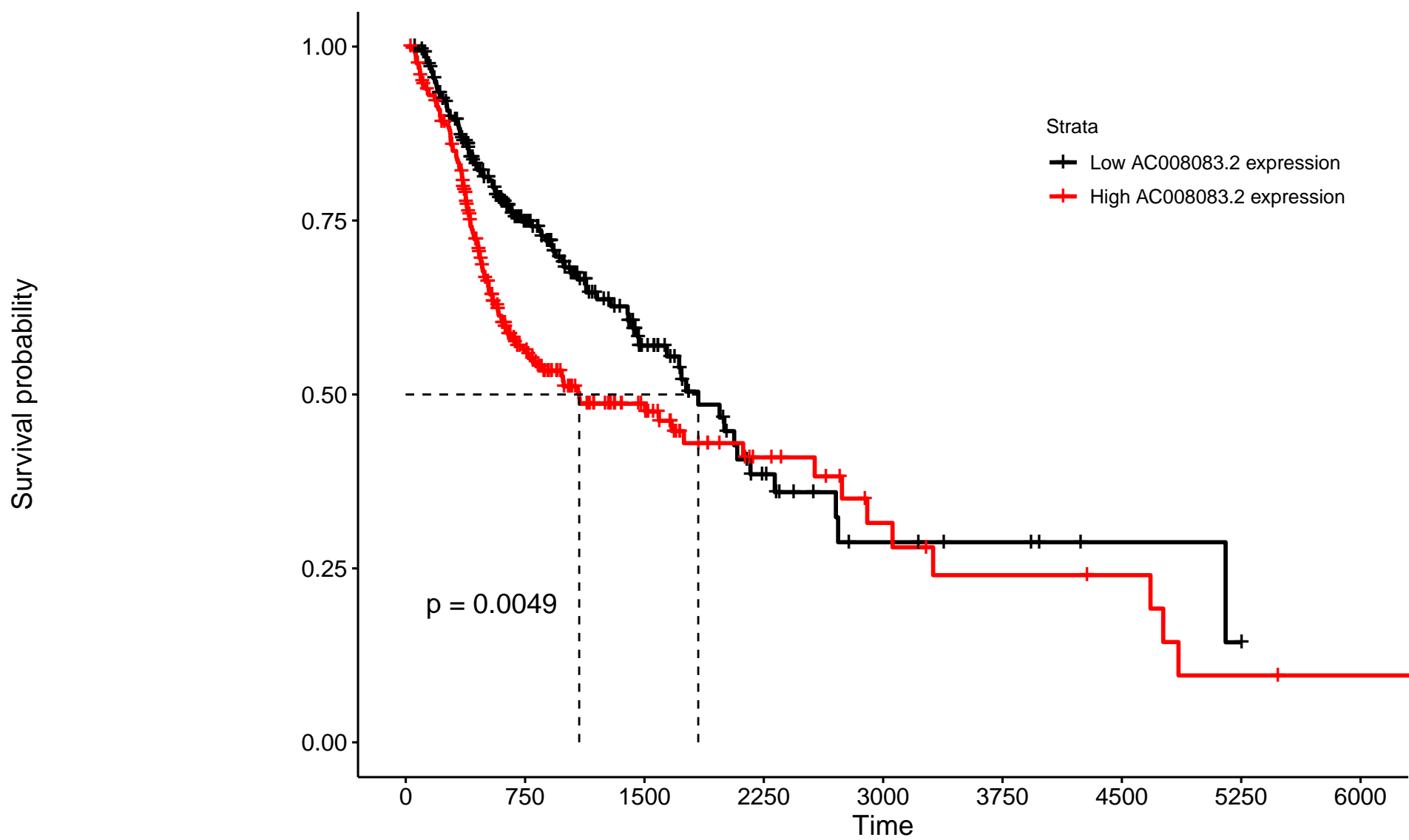

Number at risk

|                            |     |     |      |      |      |      |      |      |      |
|----------------------------|-----|-----|------|------|------|------|------|------|------|
| Strata                     | 0   | 750 | 1500 | 2250 | 3000 | 3750 | 4500 | 5250 | 6000 |
| Low AC008083.2 expression  | 246 | 118 | 43   | 16   | 7    | 5    | 2    | 1    | 0    |
| High AC008083.2 expression | 246 | 97  | 43   | 17   | 9    | 6    | 5    | 2    | 1    |

Time

Supplement: Data S1 [file peerj-12-17859-s007.zip › Figure4/B/B1 AC008083.2.pdf]

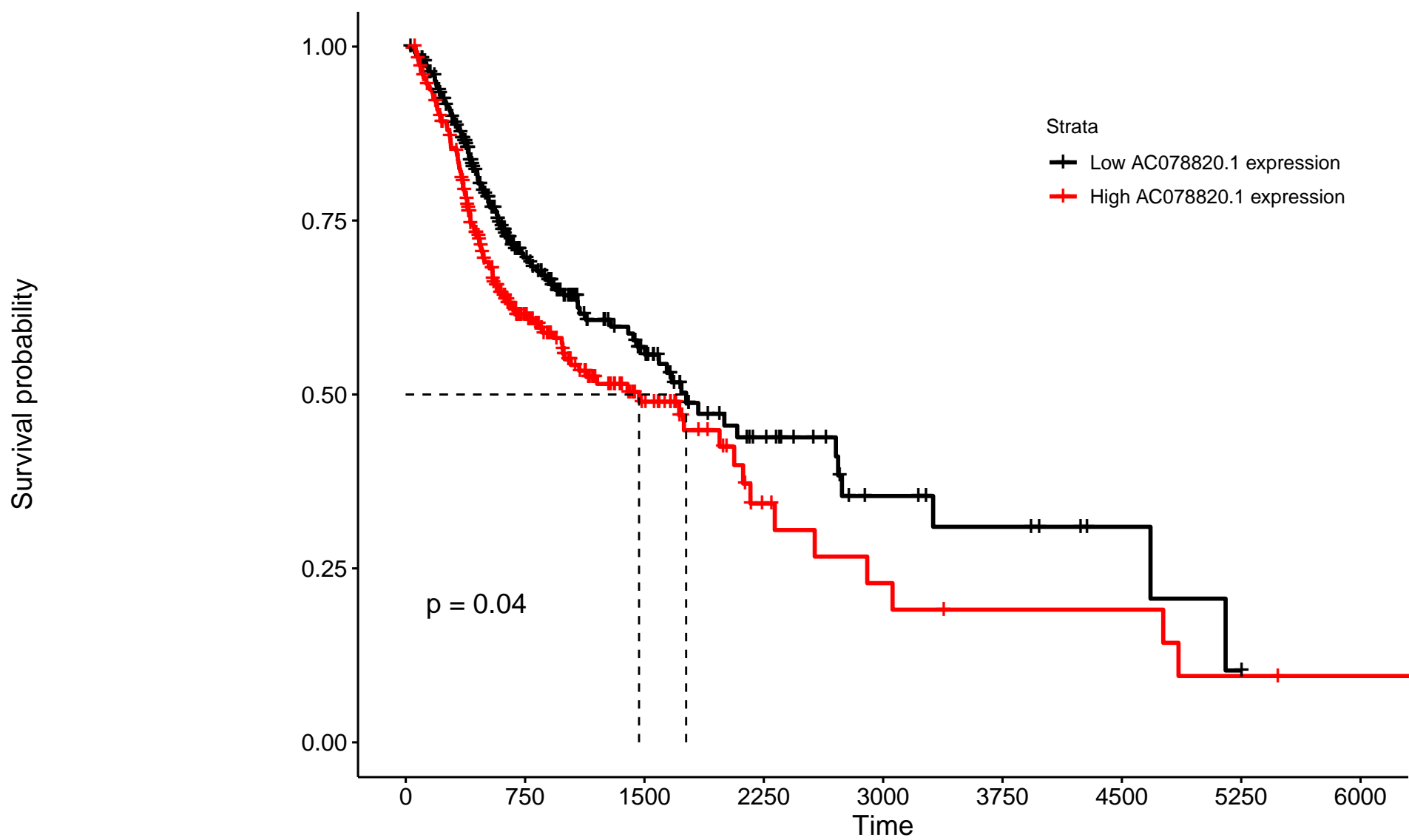

Number at risk

|                            |     |     |      |      |      |      |      |      |      |
|----------------------------|-----|-----|------|------|------|------|------|------|------|
| Strata                     | 0   | 750 | 1500 | 2250 | 3000 | 3750 | 4500 | 5250 | 6000 |
| Low AC078820.1 expression  | 246 | 113 | 52   | 23   | 10   | 7    | 3    | 1    | 0    |
| High AC078820.1 expression | 246 | 102 | 34   | 10   | 6    | 4    | 4    | 2    | 1    |

Time

Supplement: Data S1 [file peerj-12-17859-s007.zip › Figure4/B/B2 AC078820.1.pdf]

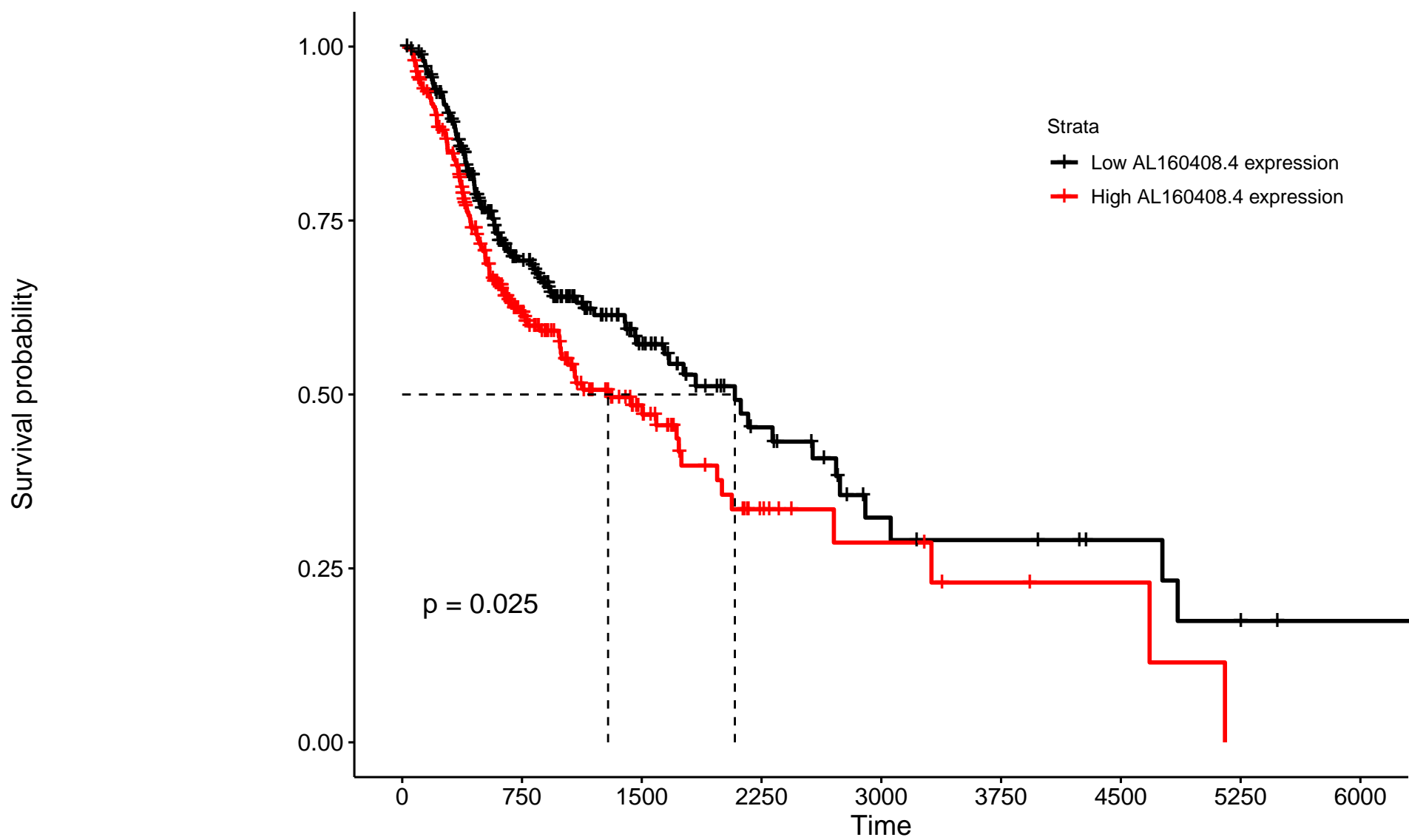

Number at risk

| Strata                     | 0   | 750 | 1500 | 2250 | 3000 | 3750 | 4500 | 5250 | 6000 |
|----------------------------|-----|-----|------|------|------|------|------|------|------|
| Low AL160408.4 expression  | 246 | 117 | 50   | 22   | 10   | 8    | 5    | 3    | 1    |
| High AL160408.4 expression | 246 | 98  | 36   | 11   | 6    | 3    | 2    | 0    | 0    |

Time

Supplement: Data S1 [file peerj-12-17859-s007.zip › Figure4/B/B3 AL160408.4.pdf]

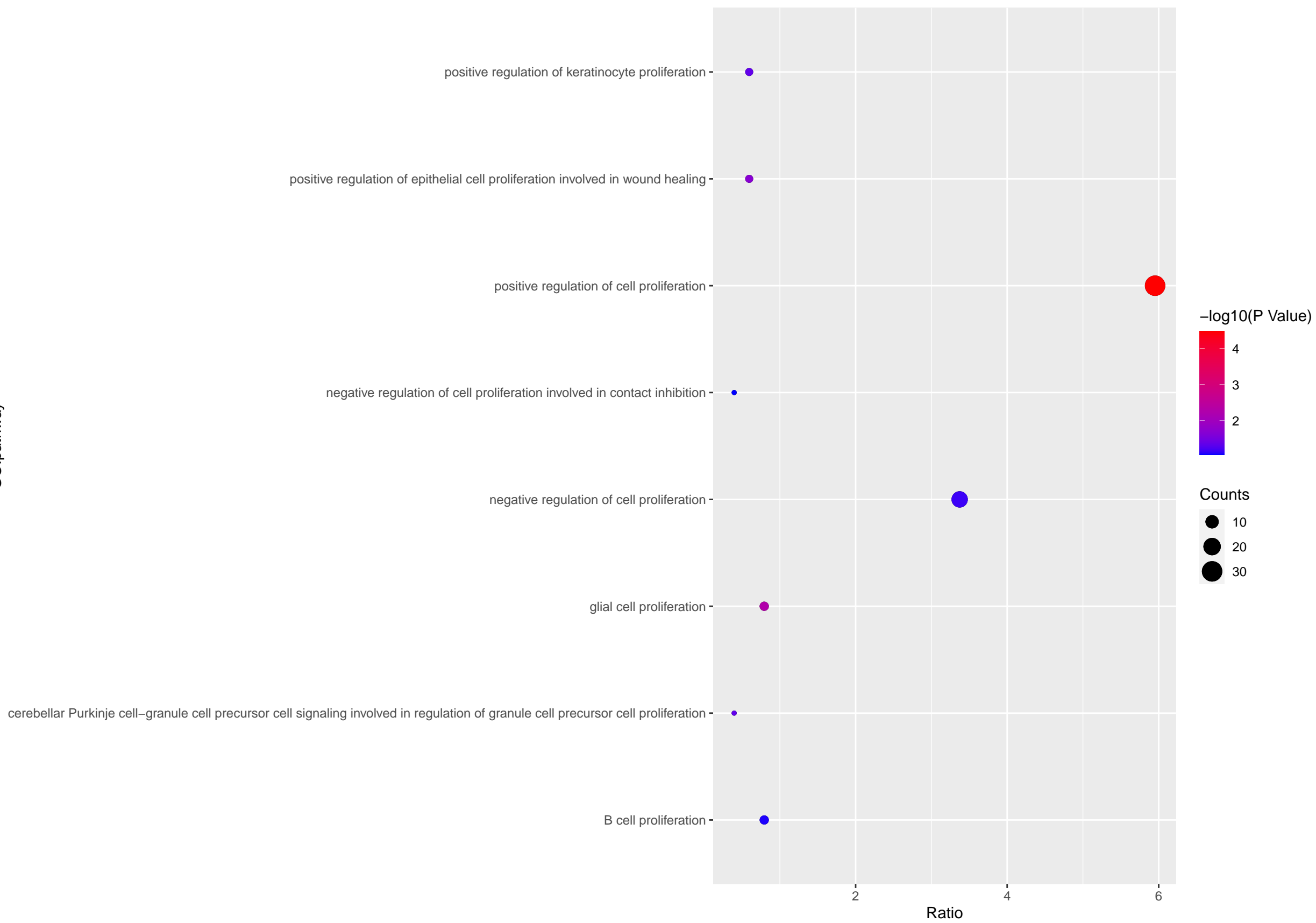

Supplement: Data S1 [file peerj-12-17859-s007.zip › Figure5/A/A1 AC008083.2.pdf]

GO.pathway

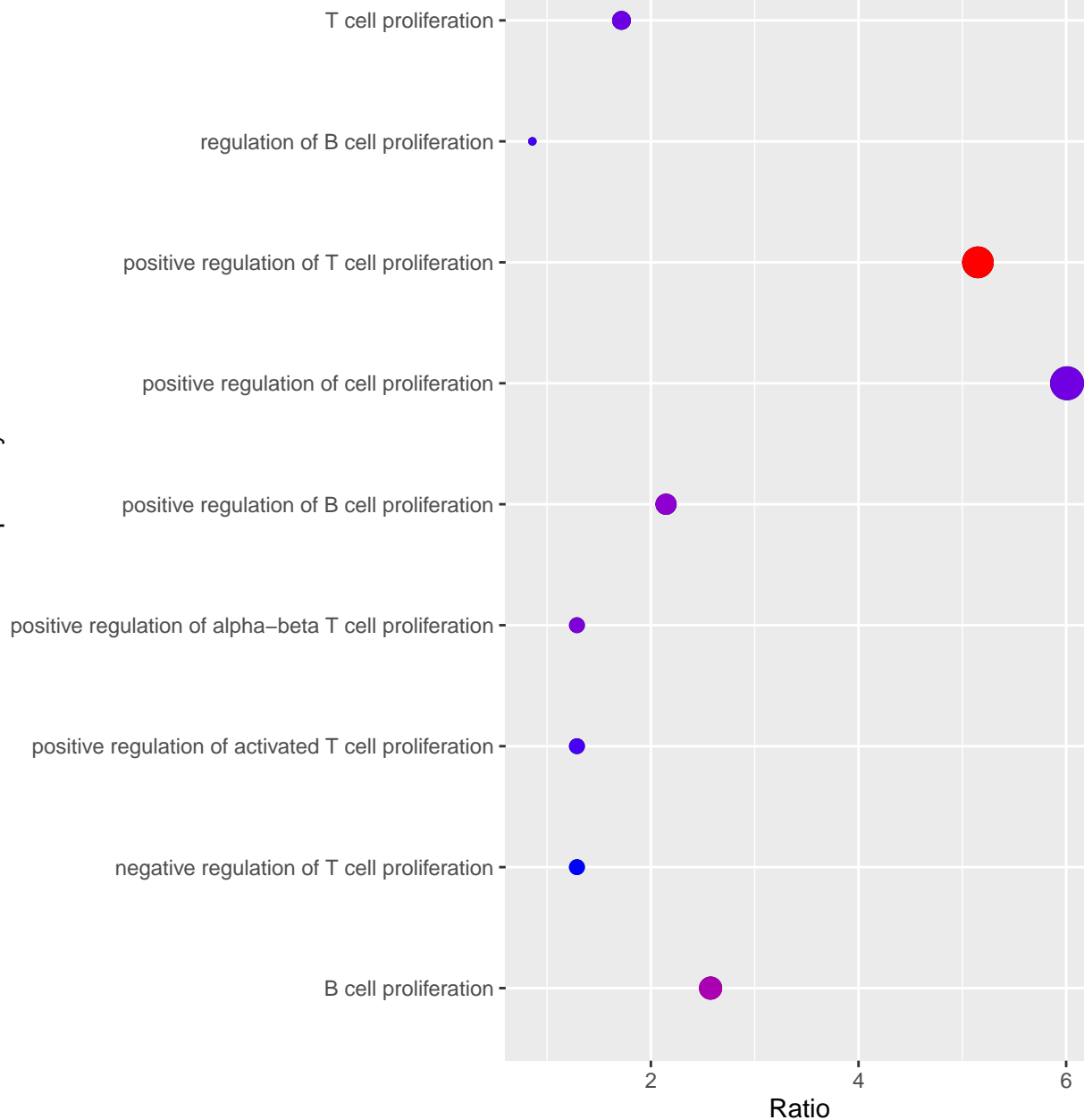

Supplement: Data S1 [file peerj-12-17859-s007.zip › Figure5/A/A2 miR−142−3p.pdf]

GO.pathway

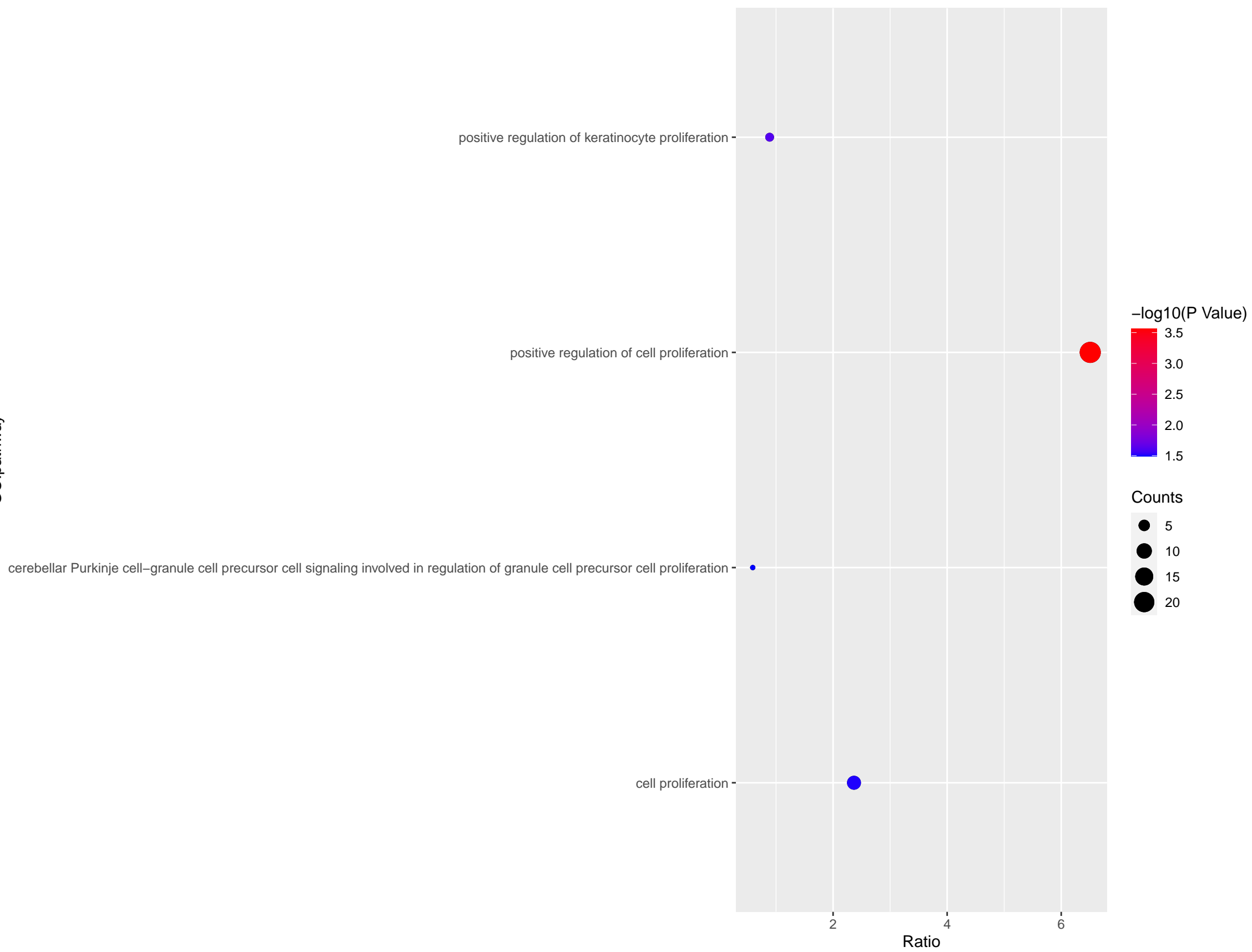

Supplement: Data S1 [file peerj-12-17859-s007.zip › Figure5/A/A3 STRN3.pdf]

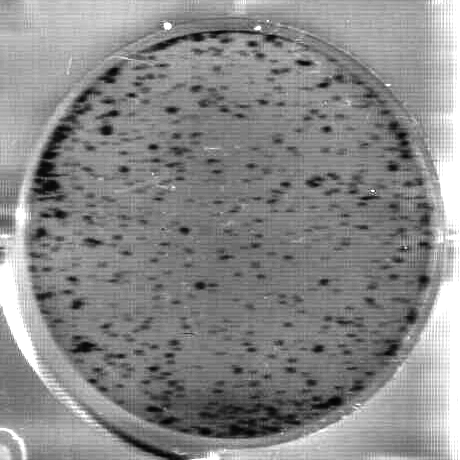

Supplement: Data S1 [file peerj-12-17859-s007.zip › Figure5/C/lncRNA/ASO AC008083.2.tif]

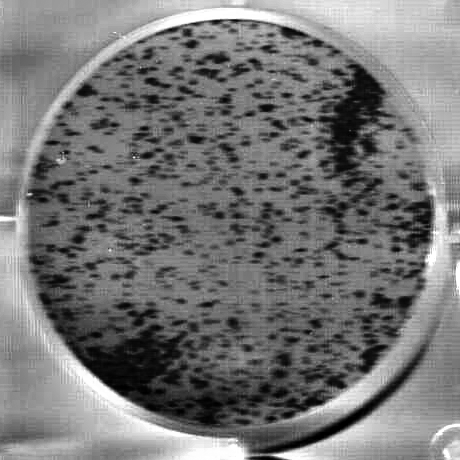

Supplement: Data S1 [file peerj-12-17859-s007.zip › Figure5/C/lncRNA/ASO NC.tif]

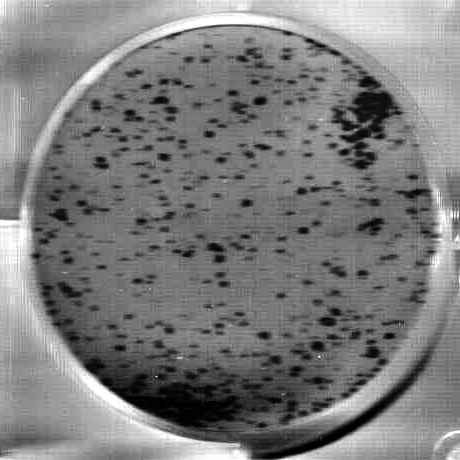

Supplement: Data S1 [file peerj-12-17859-s007.zip › Figure5/C/miRNA/miR-142-3p inhibitor.tif]

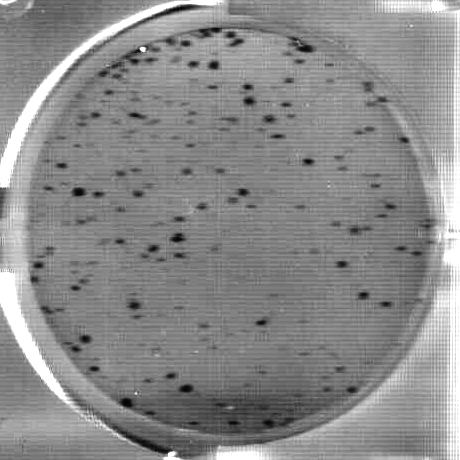

Supplement: Data S1 [file peerj-12-17859-s007.zip › Figure5/C/miRNA/NC inhibitor.tif]

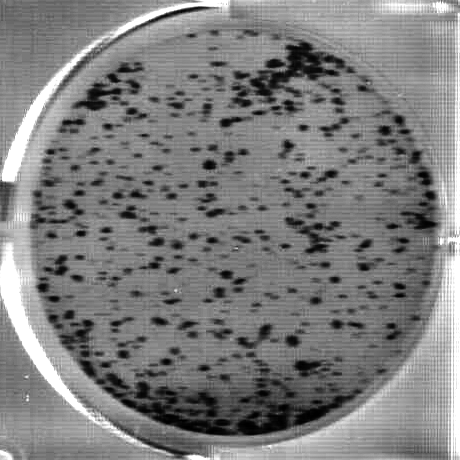

Supplement: Data S1 [file peerj-12-17859-s007.zip › Figure5/C/mRNA/shNC.tif]

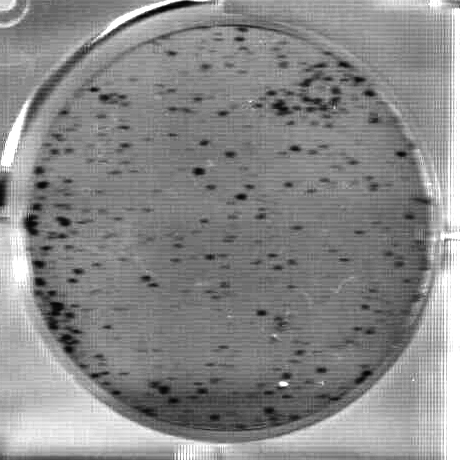

Supplement: Data S1 [file peerj-12-17859-s007.zip › Figure5/C/mRNA/shSTRN3-1.tif]

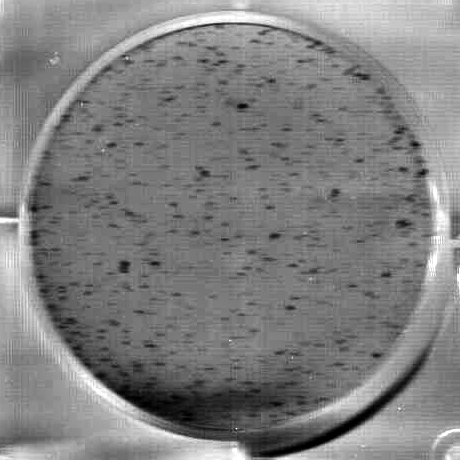

Supplement: Data S1 [file peerj-12-17859-s007.zip › Figure5/C/mRNA/shSTRN3-2.tif]

Figure6A

1. STRN3 87kda


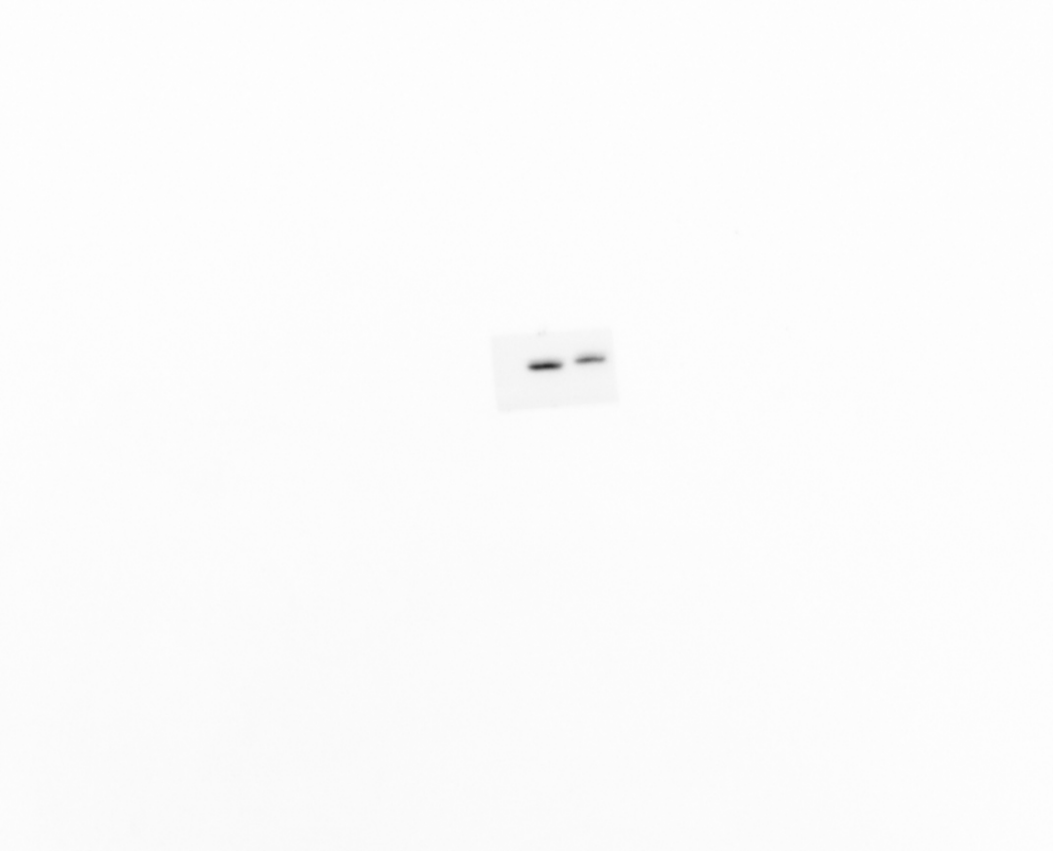


1. ACTB 43kda


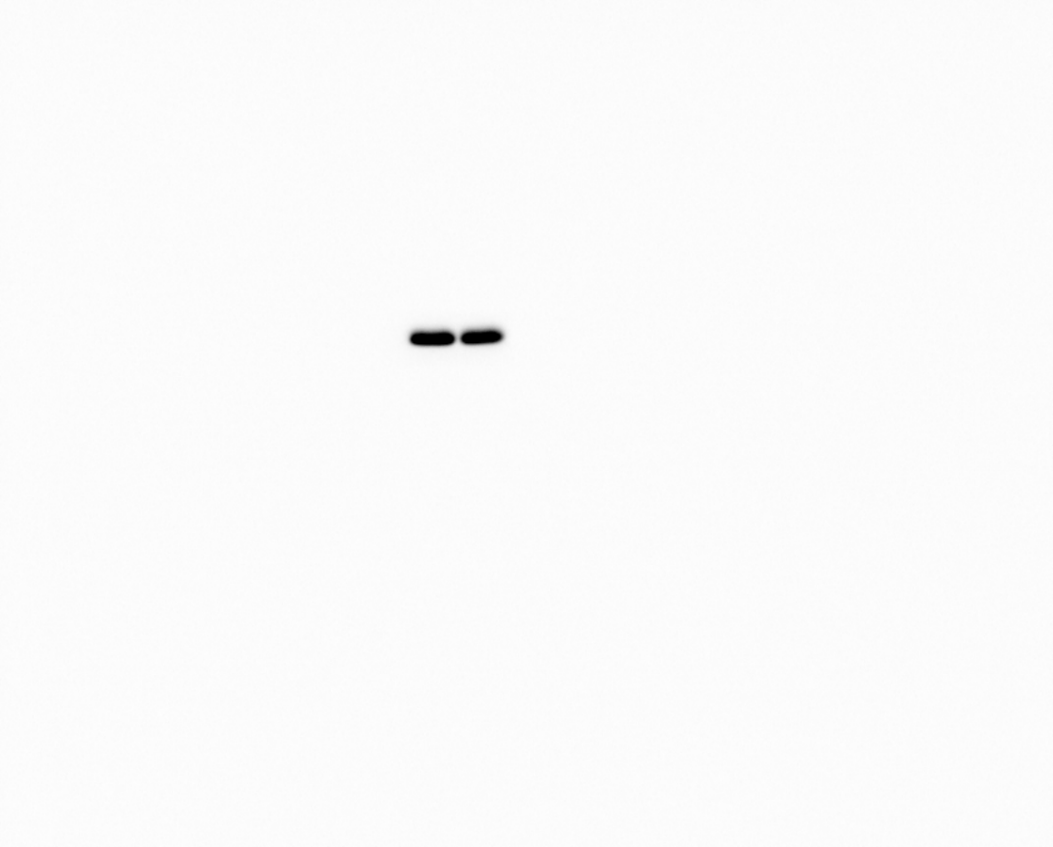


Figure6B

1. STRN3 87kda


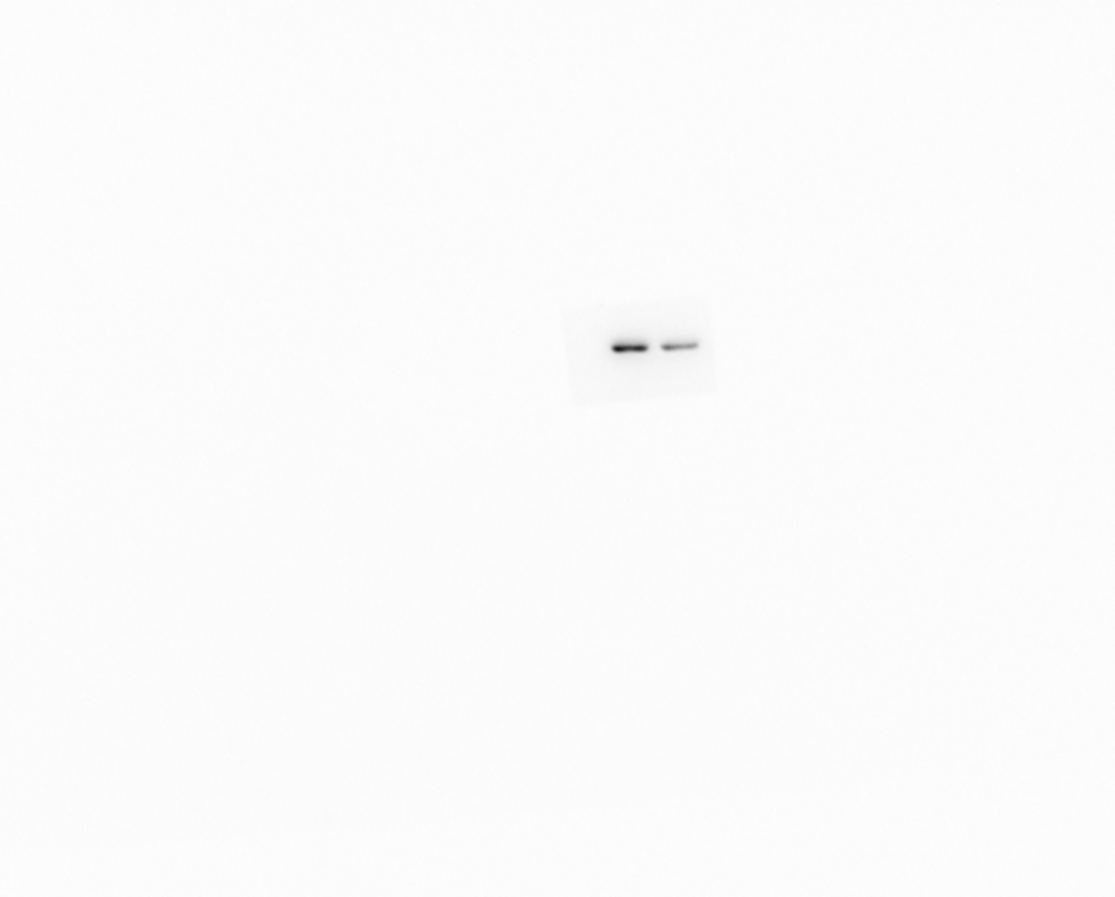


1. ACTB 43kda


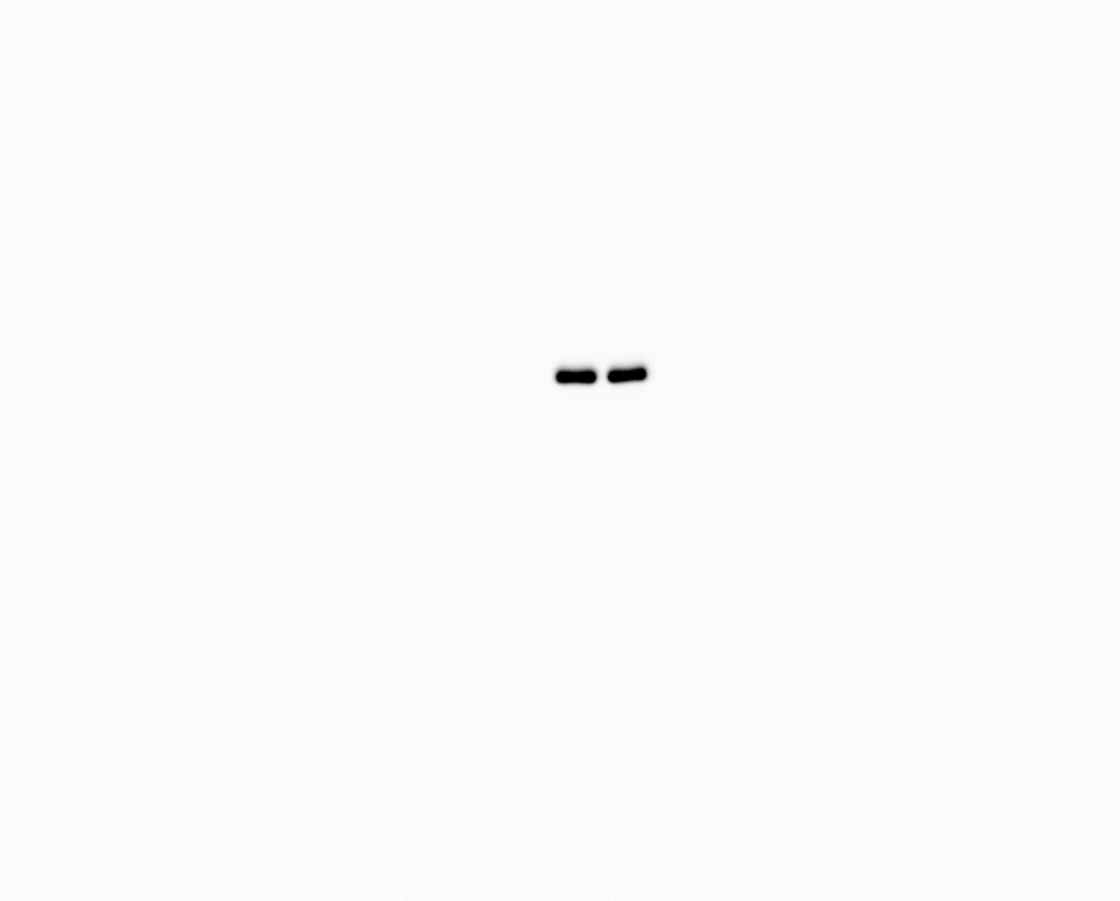


Figure6C

1. STRN3 87kda


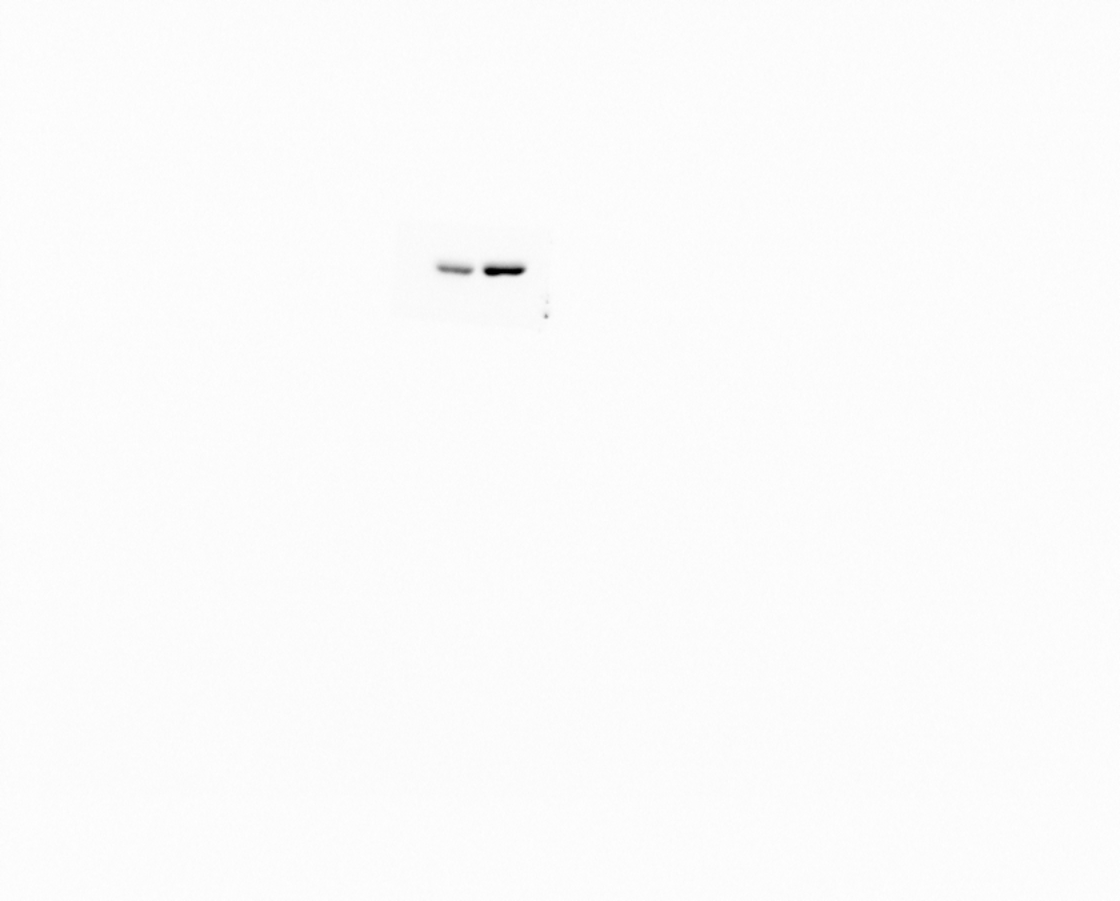


1. ACTB 43kda


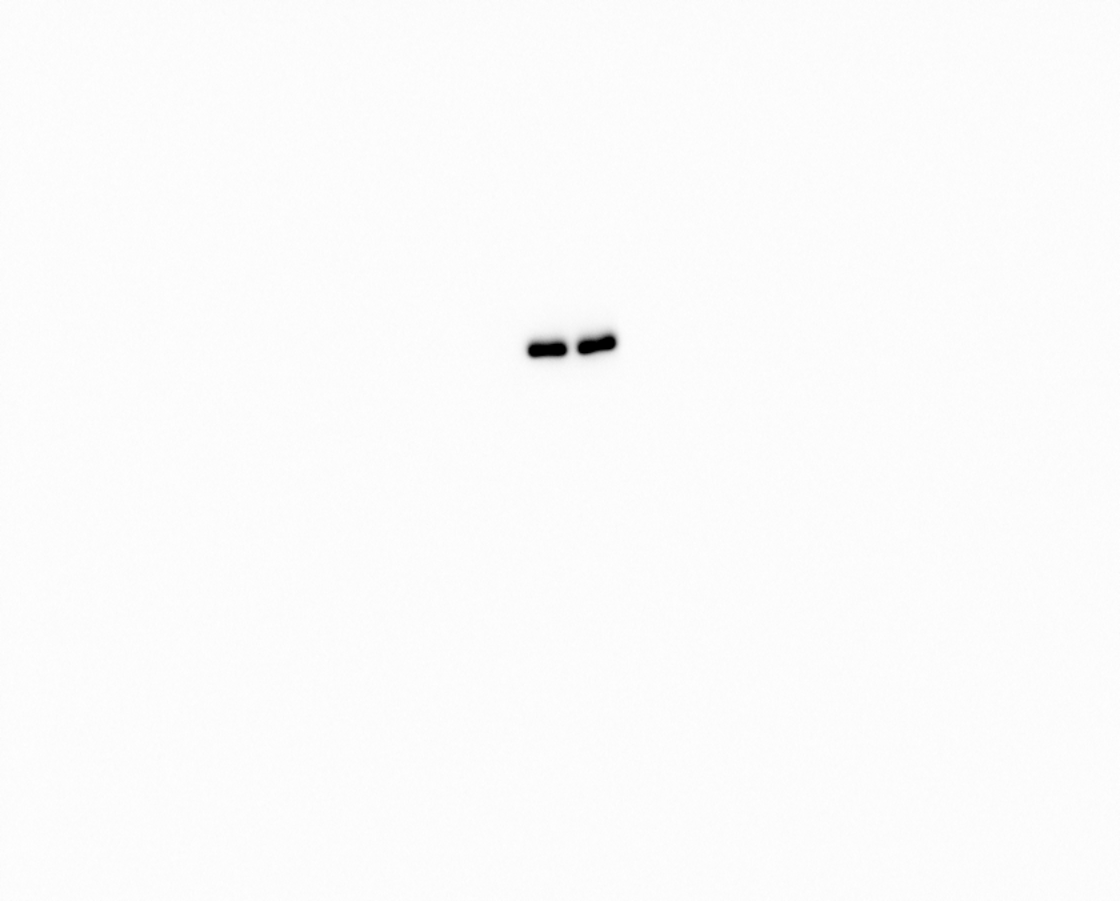

Supplement: Data S1 [file peerj-12-17859-s007.zip › Figure6/wb raw data.docx]

GO.pathway

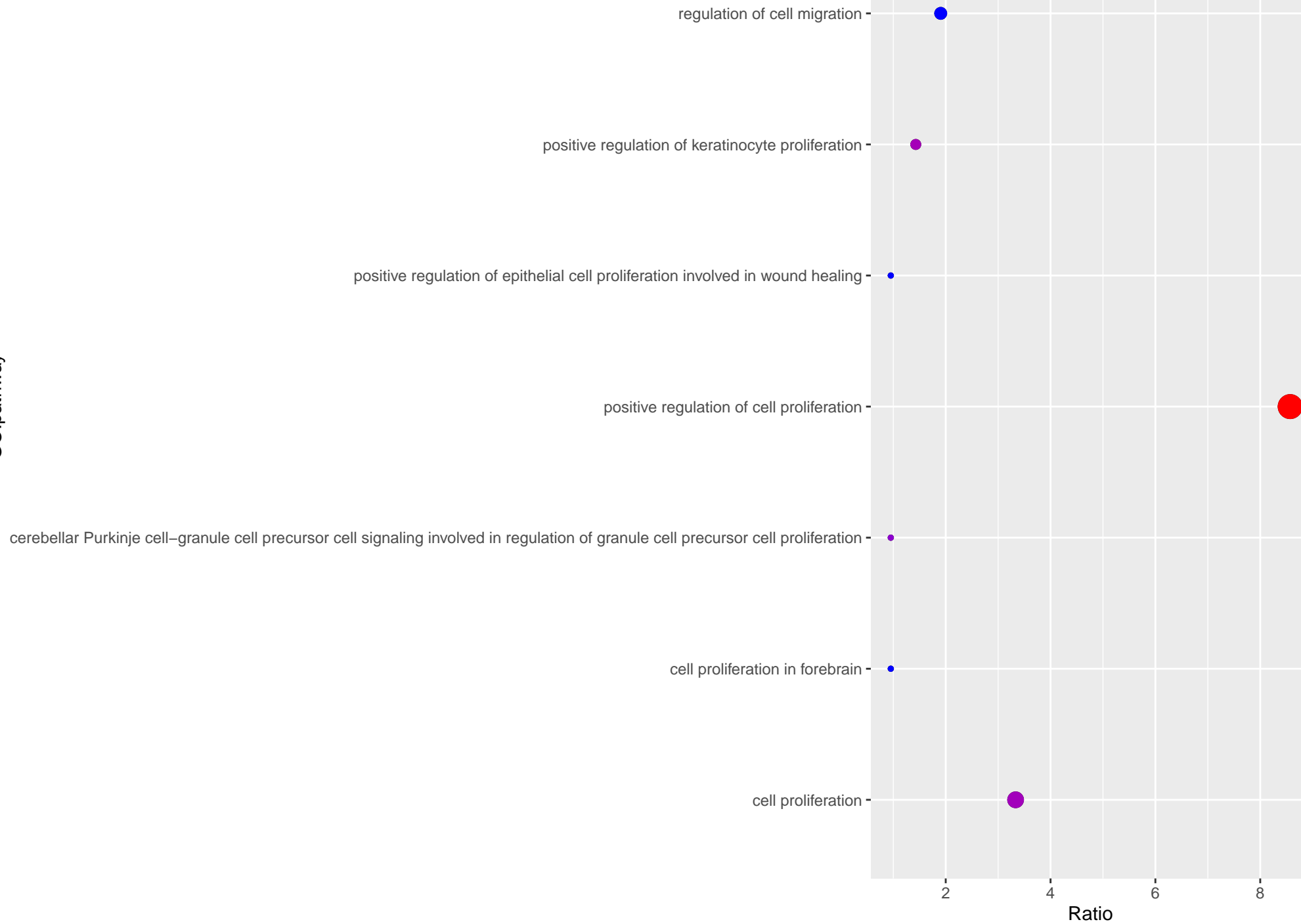

$-\log_{10}(\text{P Value})$

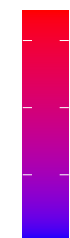

Counts

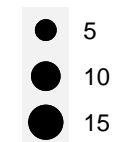

Supplement: Data S1 [file peerj-12-17859-s007.zip › Figure7/B lnc-m-bubble.pdf]

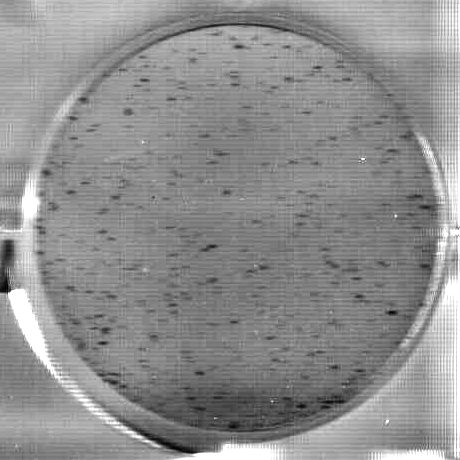

Supplement: Data S1 [file peerj-12-17859-s007.zip › Figure7/D/ASO-lncRNA+miRNA mimic.tif]

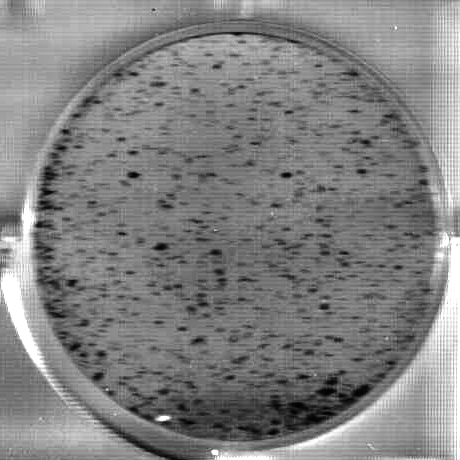

Supplement: Data S1 [file peerj-12-17859-s007.zip › Figure7/D/ASO-lncRNA+miRNA-NC.tif]

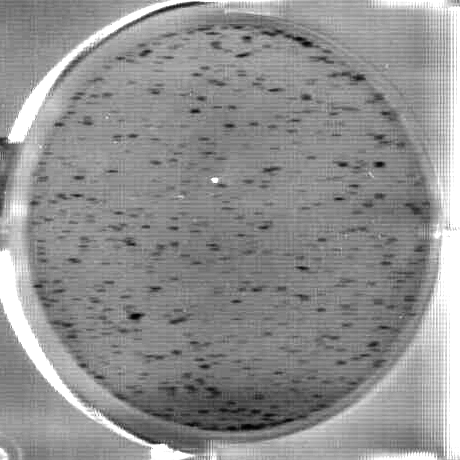

Supplement: Data S1 [file peerj-12-17859-s007.zip › Figure7/D/ASO-NC+miRNA mimic.tif]

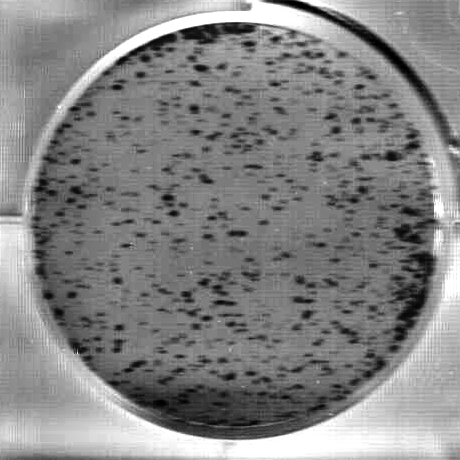

Supplement: Data S1 [file peerj-12-17859-s007.zip › Figure7/D/ASO-NC+miRNA-NC.tif]

Individuals – PCA

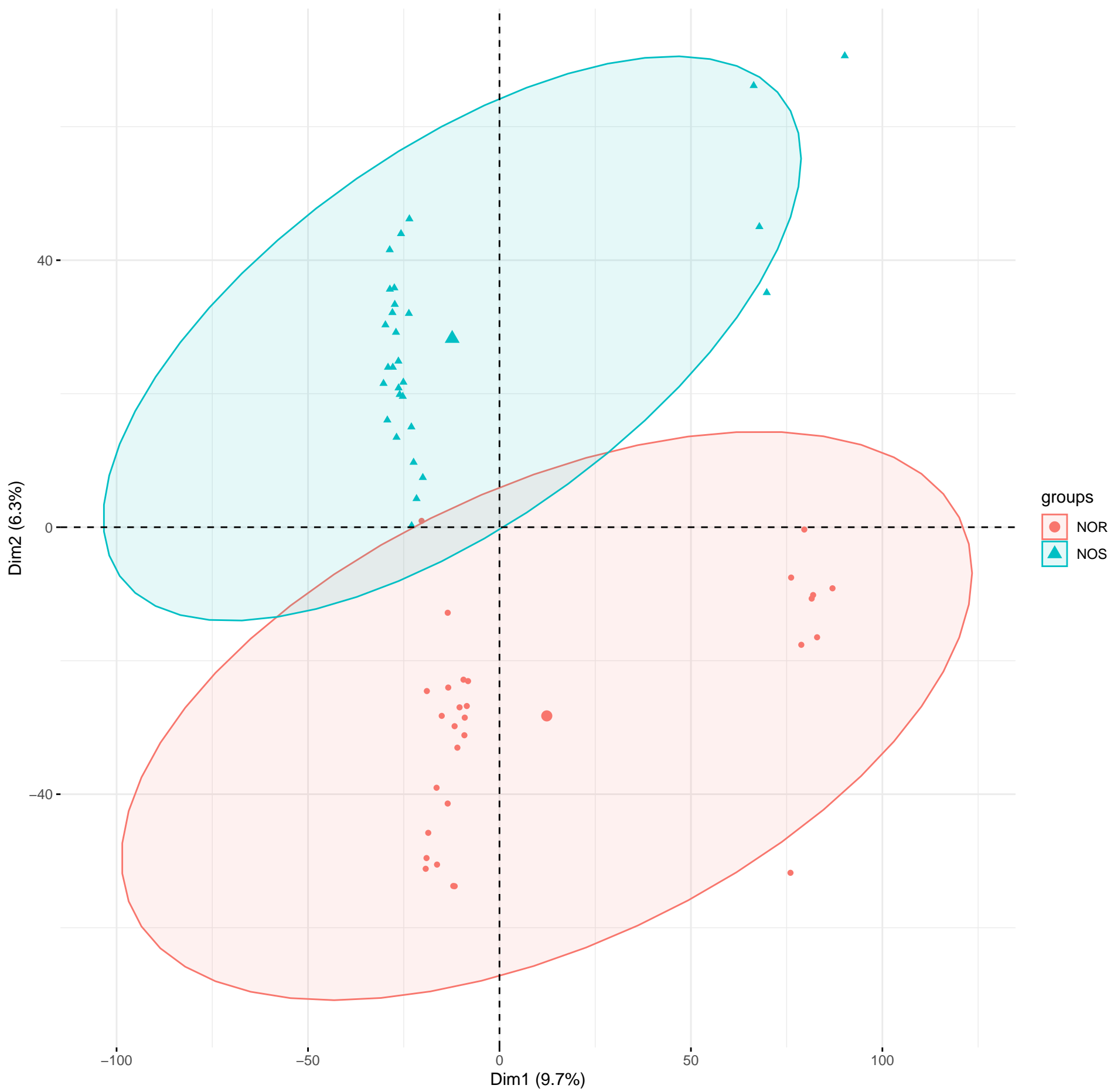

Supplement: Data S1 [file peerj-12-17859-s007.zip › FigureS1/A1 LNC pca.pdf]

Individuals – PCA

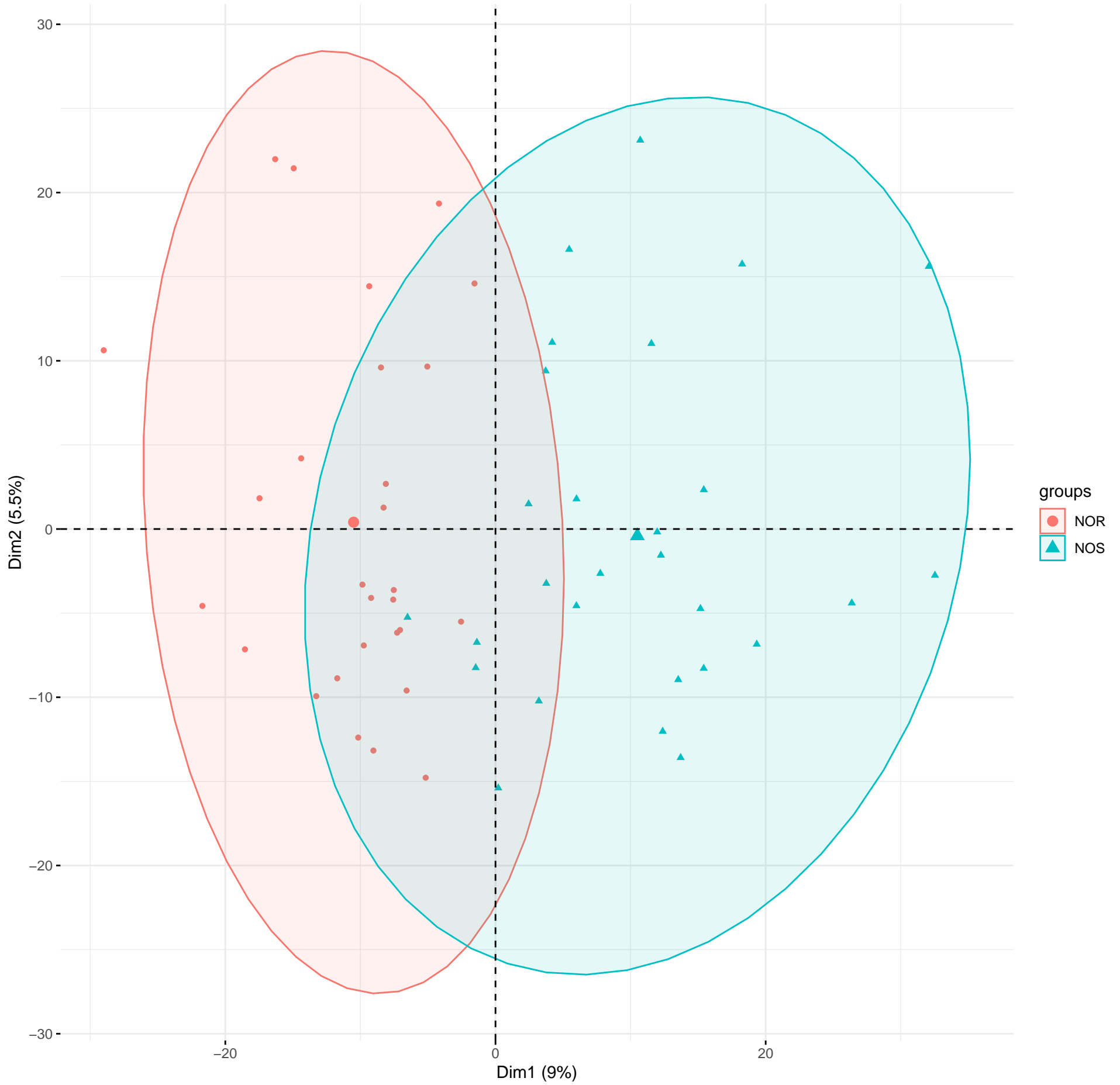

Supplement: Data S1 [file peerj-12-17859-s007.zip › FigureS1/A2 MI pca.pdf]

### consensus CDF

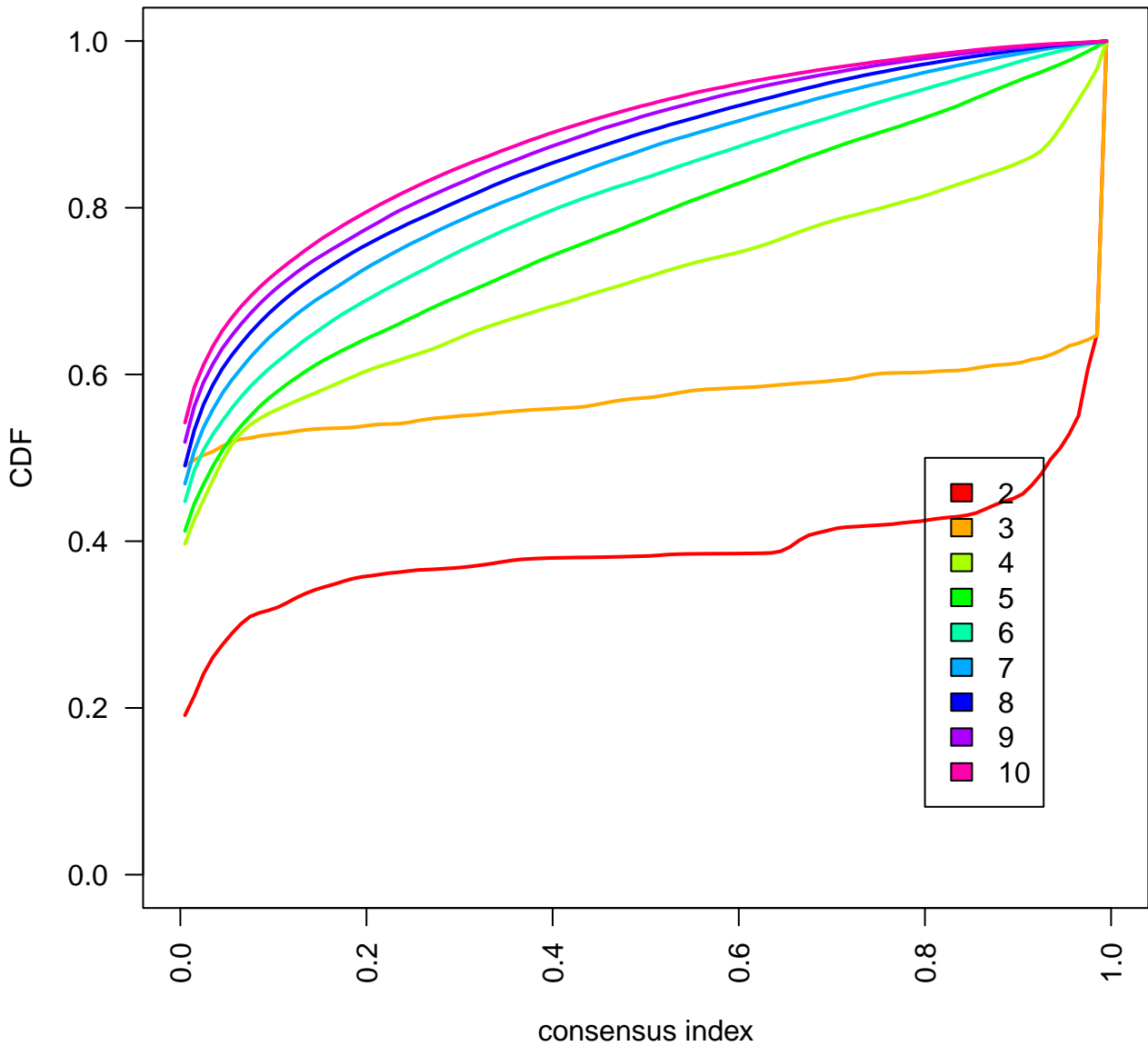

Supplement: Data S1 [file peerj-12-17859-s007.zip › FigureS2/A.pdf]

## Delta area

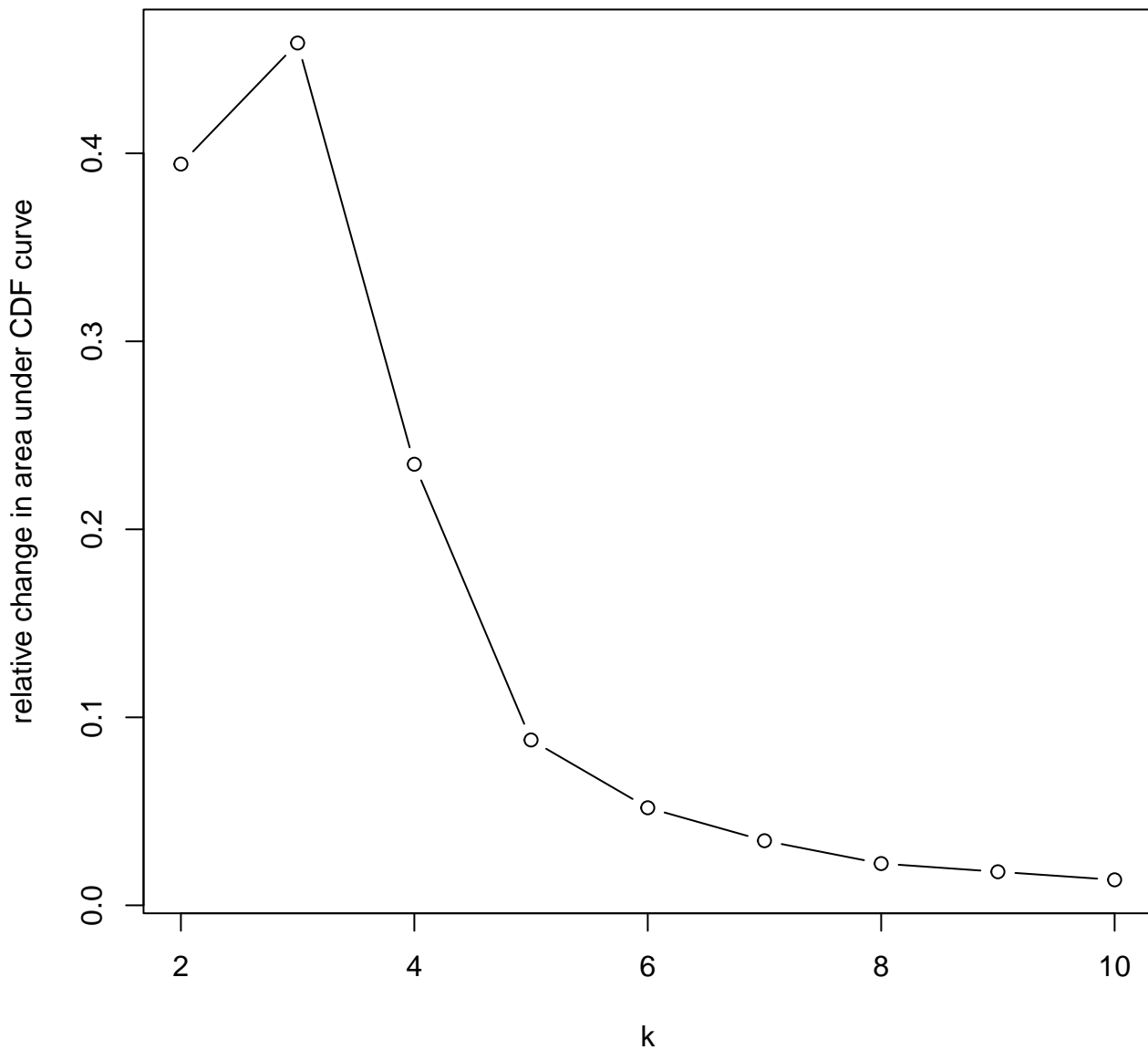

Supplement: Data S1 [file peerj-12-17859-s007.zip › FigureS2/B.pdf]

Individuals – PCA

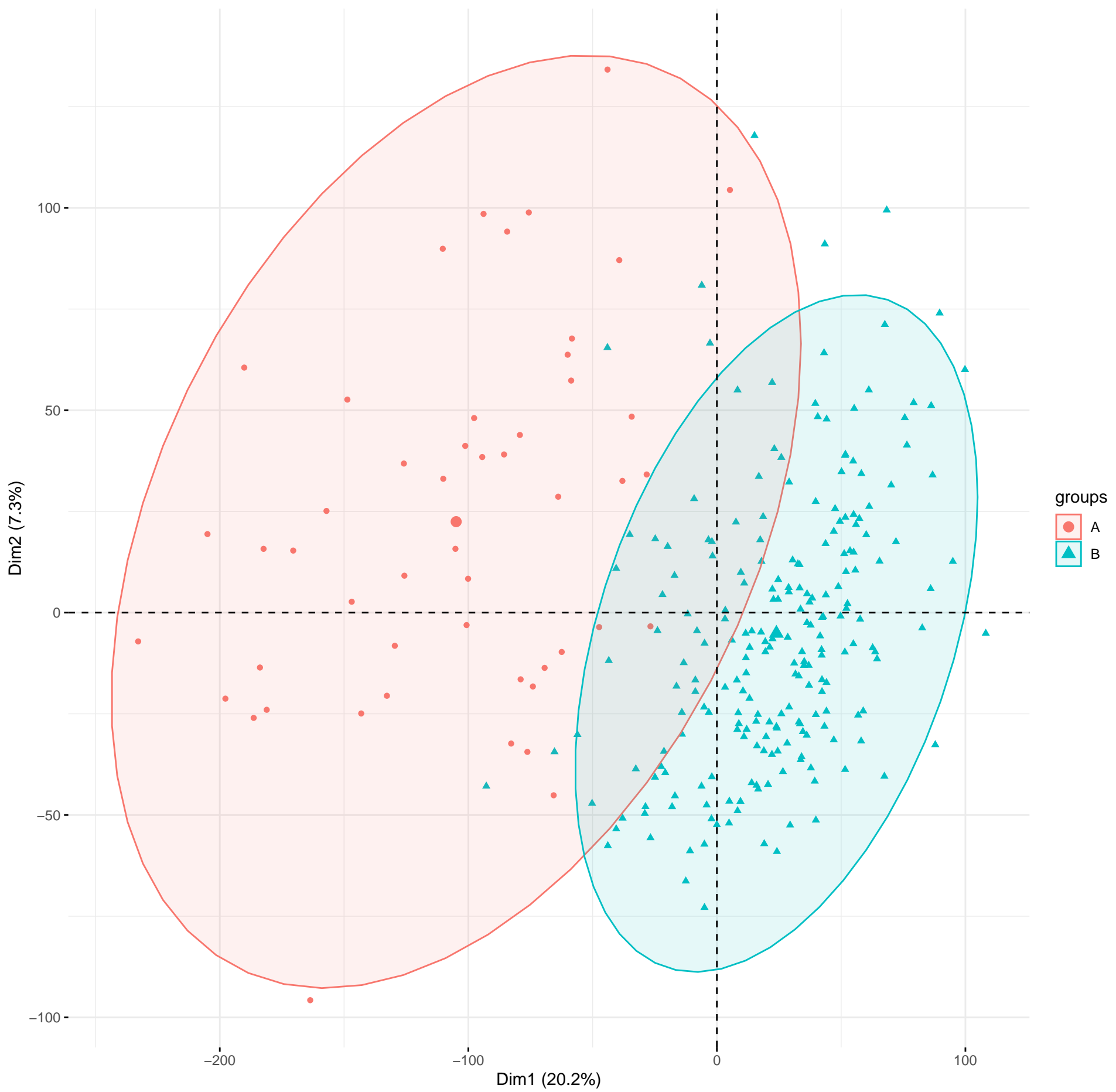

Supplement: Data S1 [file peerj-12-17859-s007.zip › FigureS2/C pca.pdf]

oncogene

up

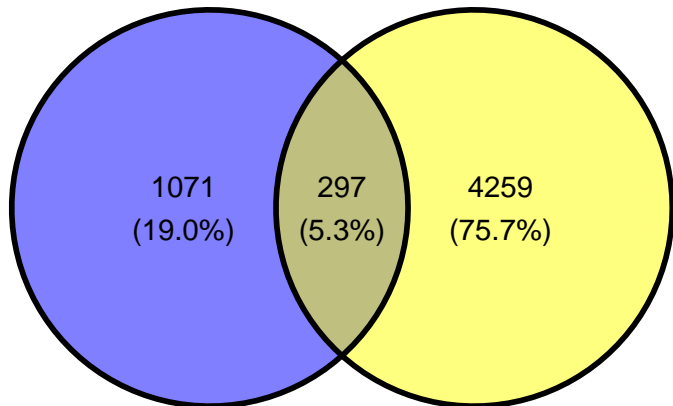

Supplement: Data S1 [file peerj-12-17859-s007.zip › FigureS2/D1 VN-1 (1).pdf]

supressgene

down

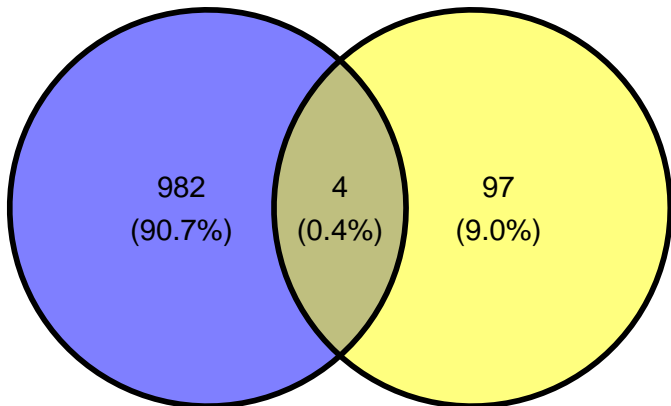

Supplement: Data S1 [file peerj-12-17859-s007.zip › FigureS2/D2 VN-1 (2).pdf]

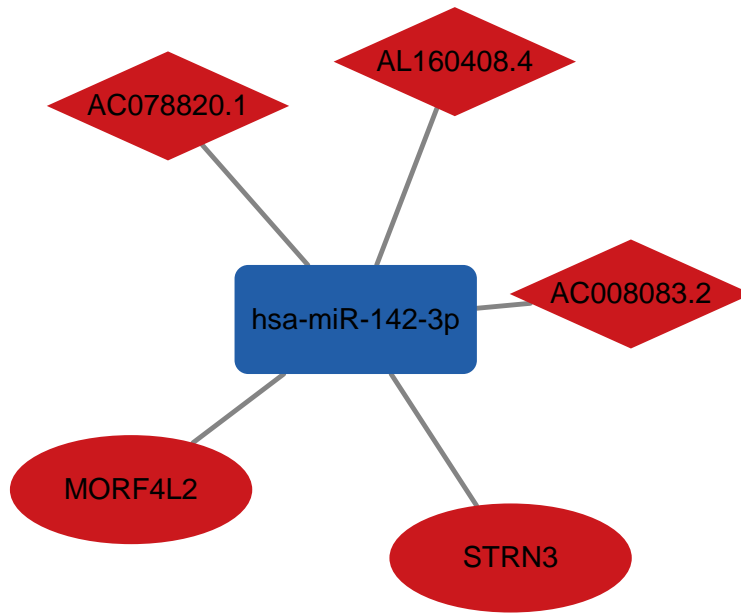

Supplement: Data S1 [file peerj-12-17859-s007.zip › FigureS3/1_TCGA_nos_ceRNA_t_a_t_interaction.txt.pdf]

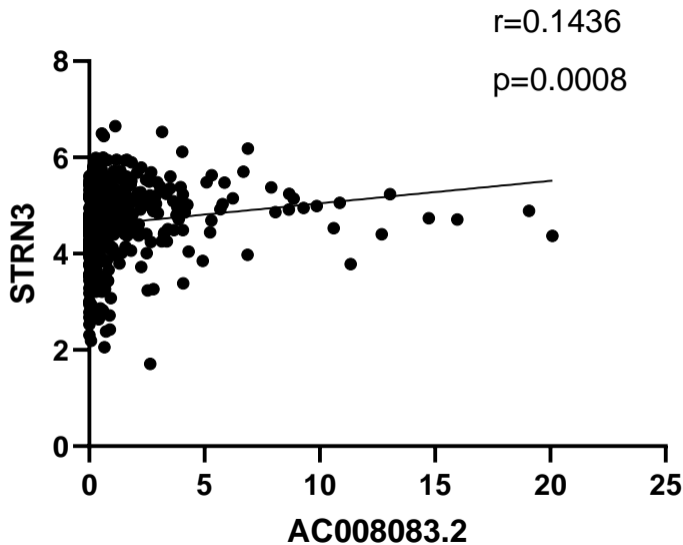

Supplement: Data S1 [file peerj-12-17859-s007.zip › FigureS4/AC008083.2.pdf]

STRN3

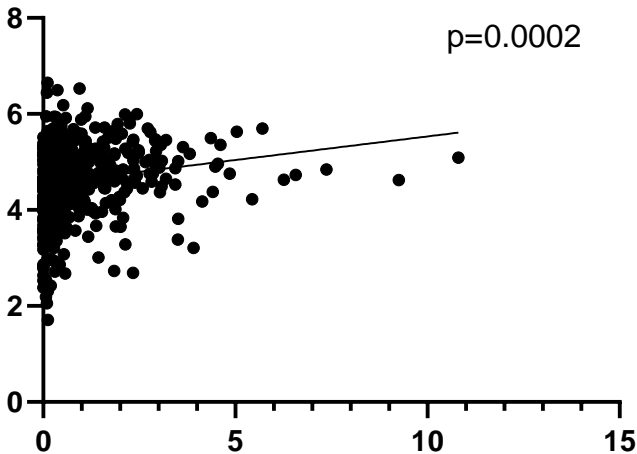

Supplement: Data S1 [file peerj-12-17859-s007.zip › FigureS4/AC078820.1.pdf]

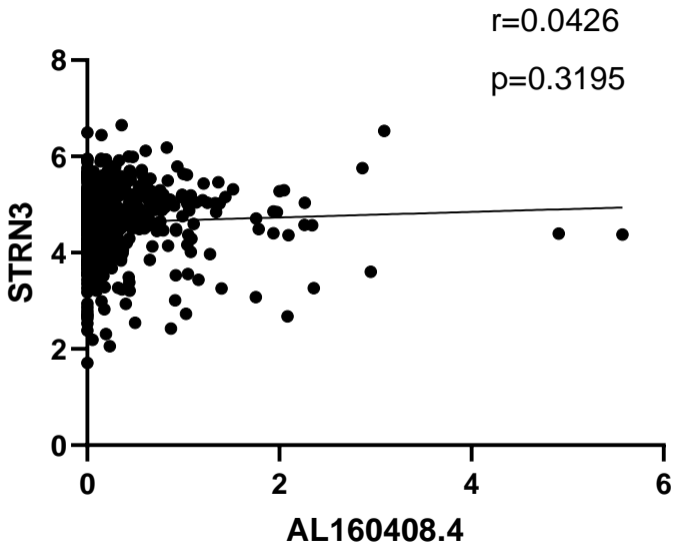

Supplement: Data S1 [file peerj-12-17859-s007.zip › FigureS4/AL160408.4.pdf]

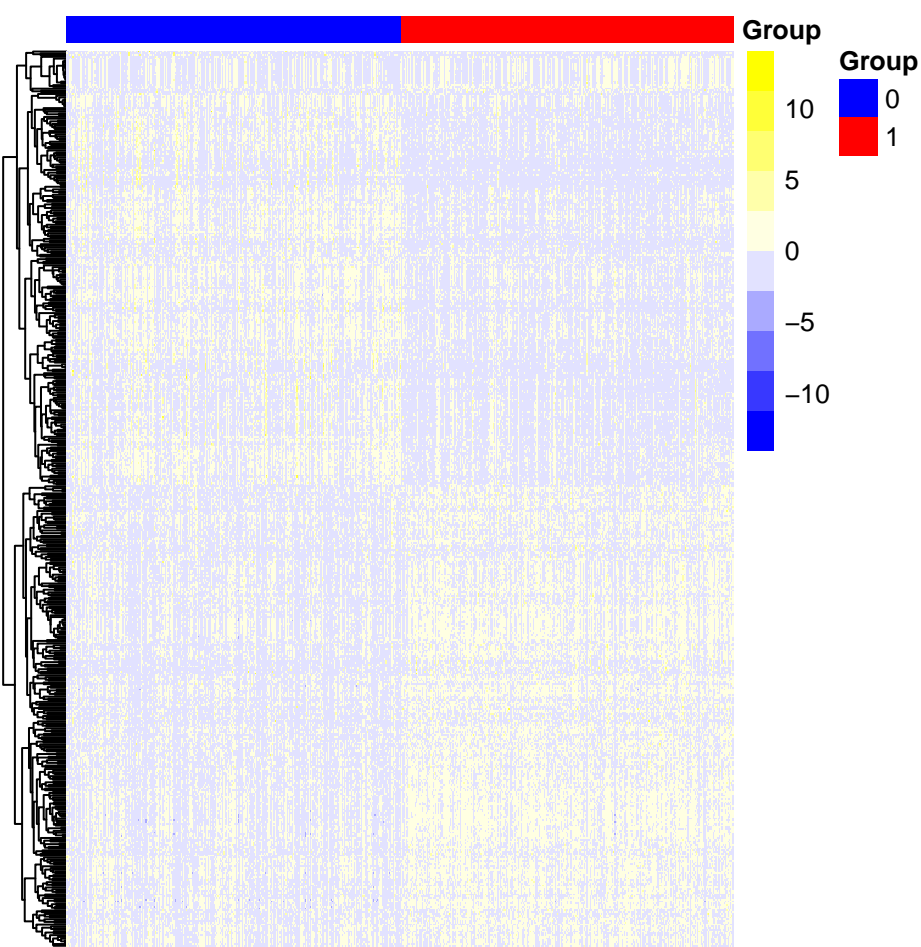

Supplement: Data S1 [file peerj-12-17859-s007.zip › FigureS5/A AC008083.2/A1 Rplot-lnc-rt.pdf]

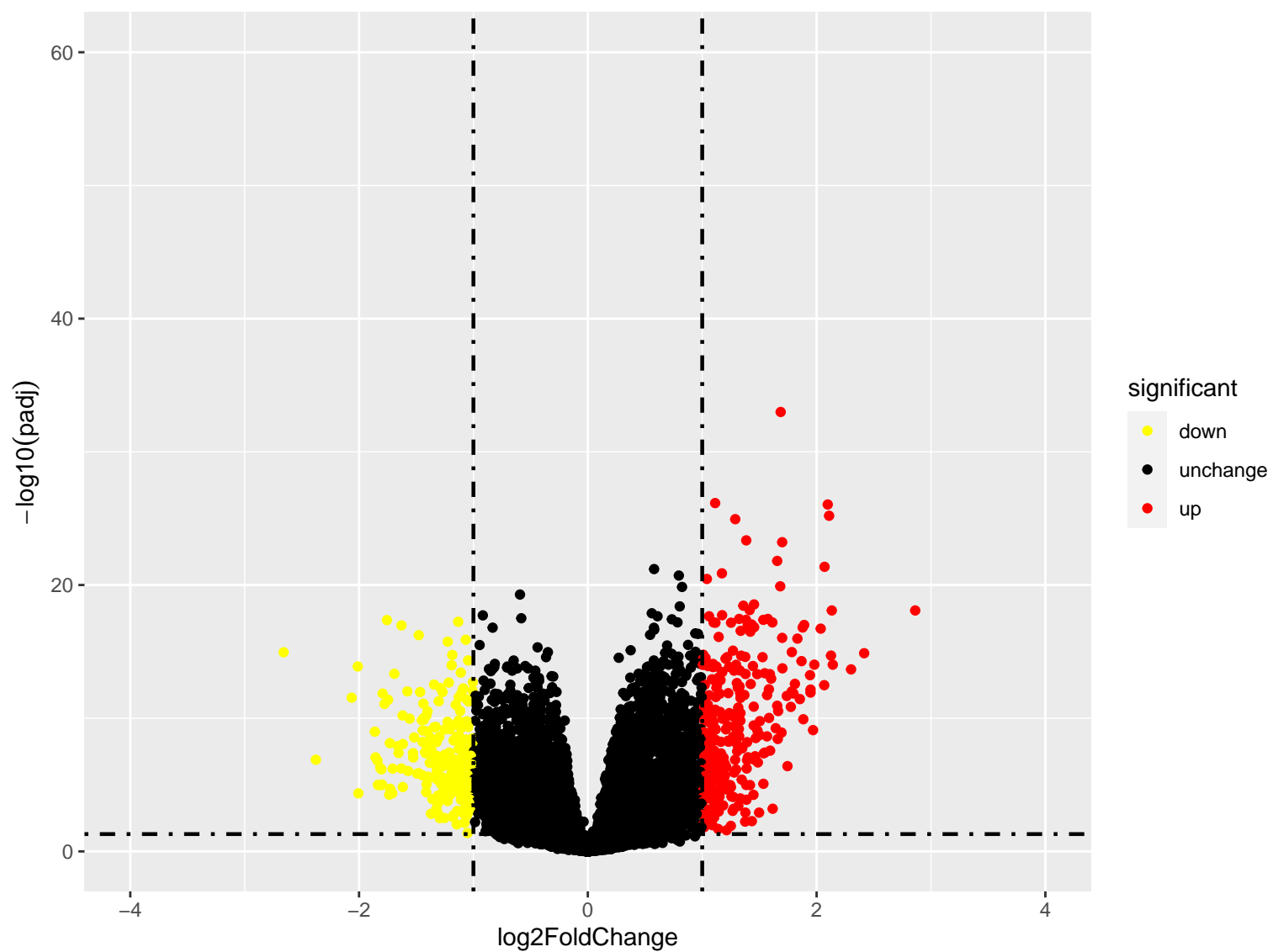

Supplement: Data S1 [file peerj-12-17859-s007.zip › FigureS5/A AC008083.2/A2 lncRNA_volcano.pdf]

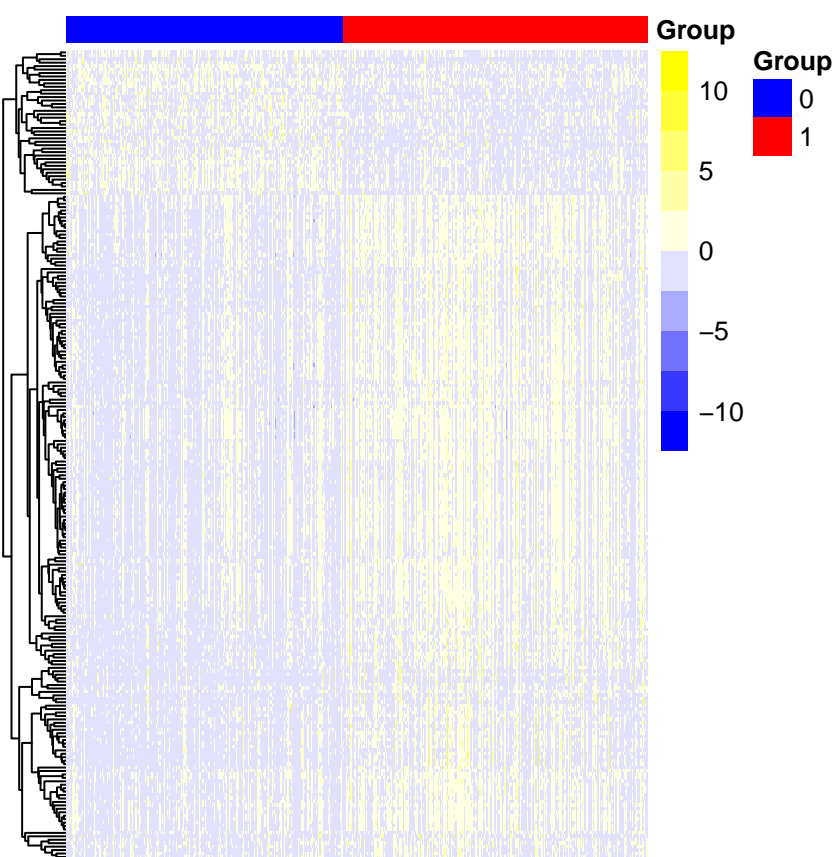

Supplement: Data S1 [file peerj-12-17859-s007.zip › FigureS5/B miR−142−3p/B1 mi-rt.pdf]

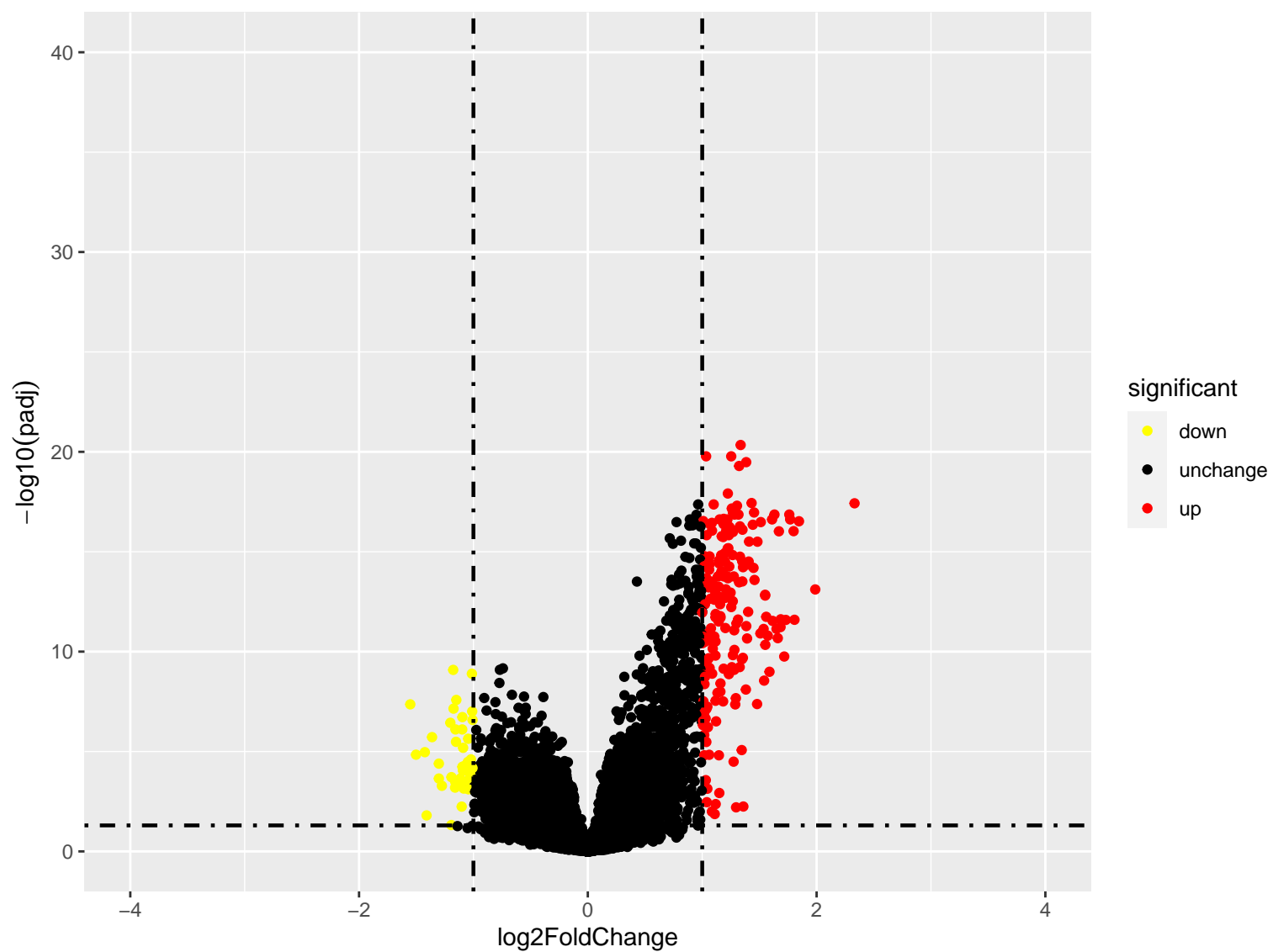

Supplement: Data S1 [file peerj-12-17859-s007.zip › FigureS5/B miR−142−3p/B2 miRNA_volcano.pdf]

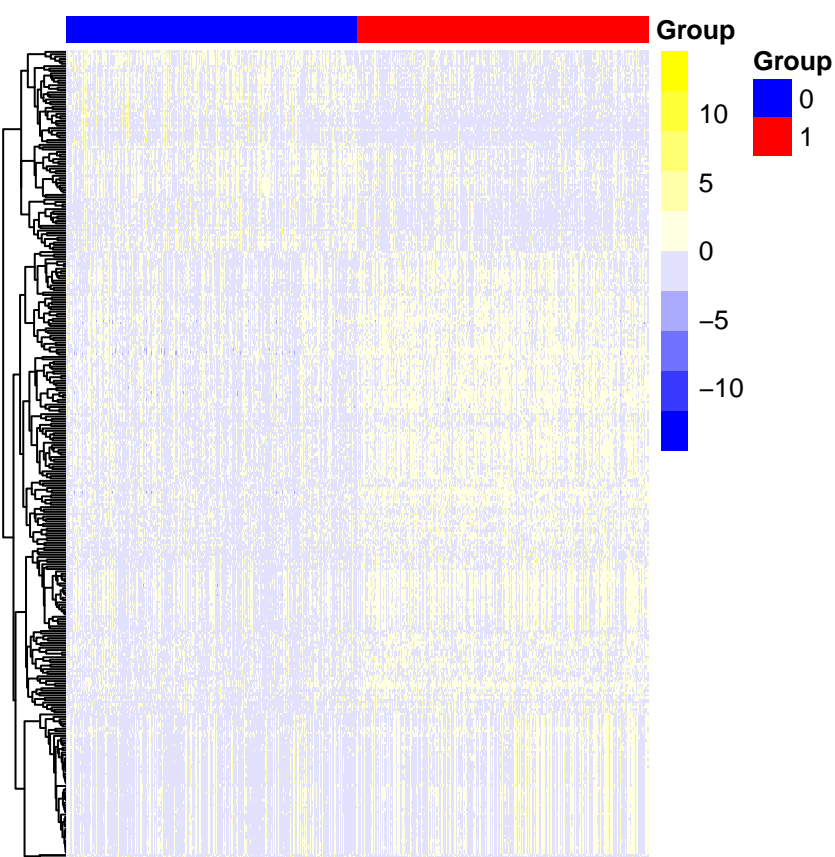

Supplement: Data S1 [file peerj-12-17859-s007.zip › FigureS5/C STRN3/C1 mRNA-RT.pdf]

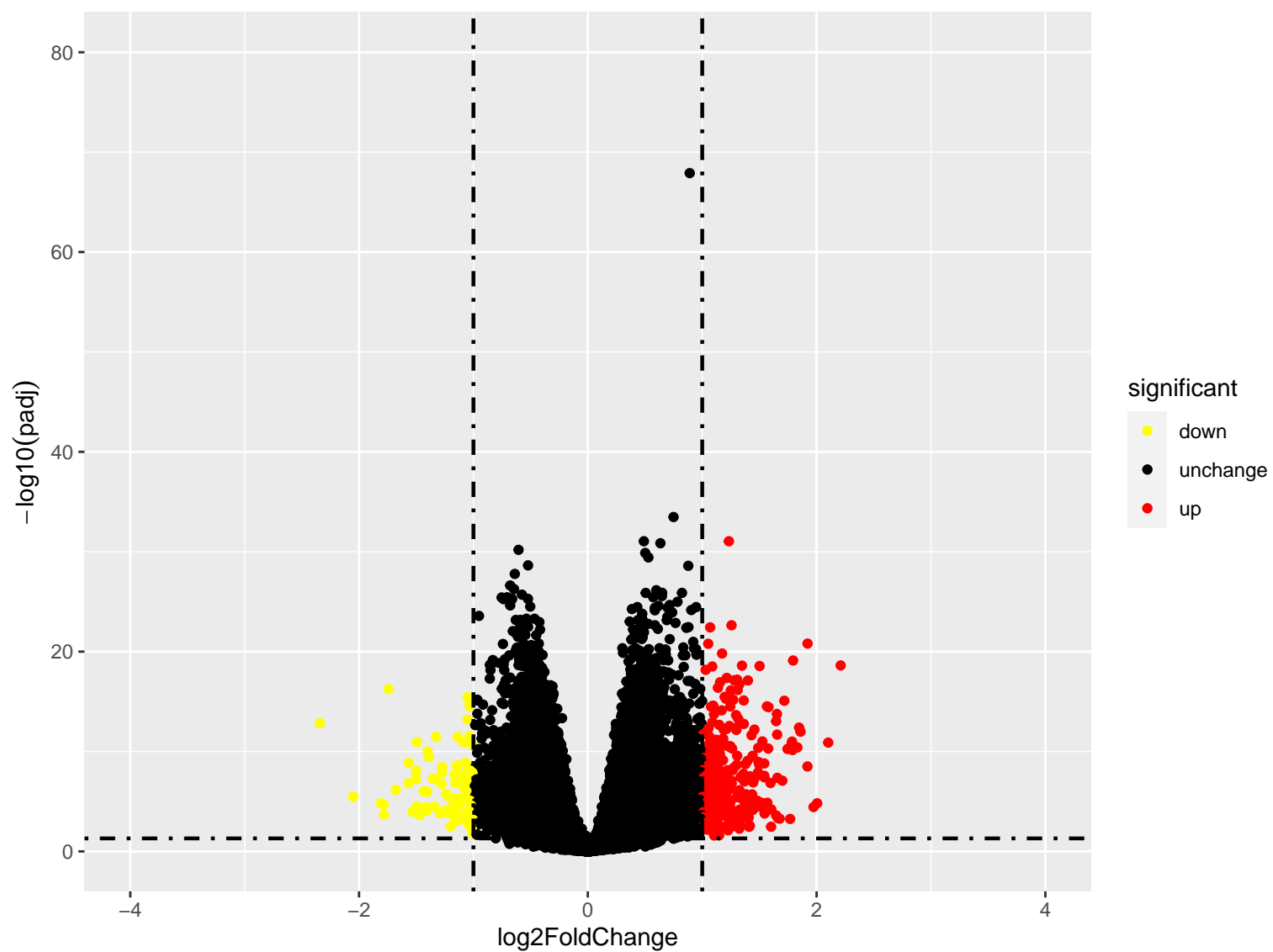

Supplement: Data S1 [file peerj-12-17859-s007.zip › FigureS5/C STRN3/C2 mRNANOS2_volcano.pdf]

lncRNA\_up

miRNA\_down

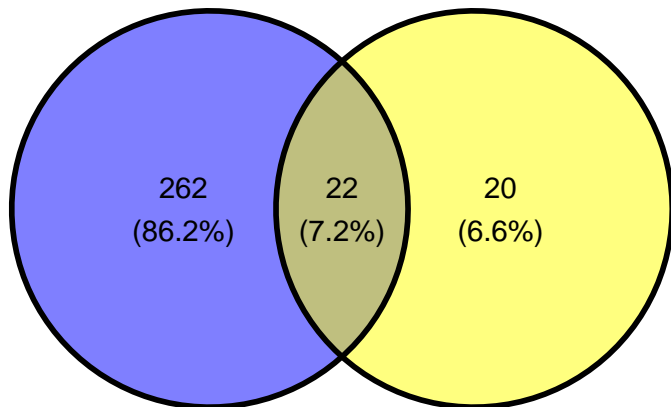

Supplement: Data S1 [file peerj-12-17859-s007.zip › FigureS6/A1 lncmi-1.pdf]

lncRNA\_down

miRNA\_up

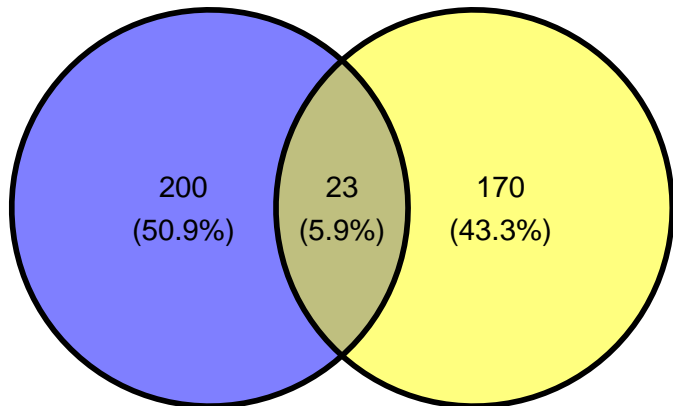

Supplement: Data S1 [file peerj-12-17859-s007.zip › FigureS6/A2 lncmi-2.pdf]

mRNA\_up

lncRNA\_up

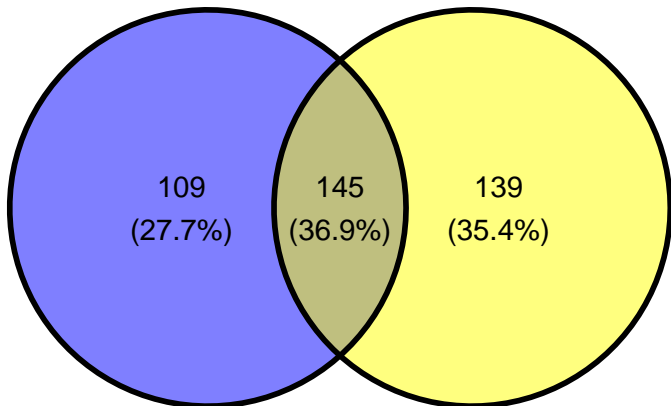

Supplement: Data S1 [file peerj-12-17859-s007.zip › FigureS6/B1 mlnc-1.pdf]

mRNA\_down    lncRNA\_down

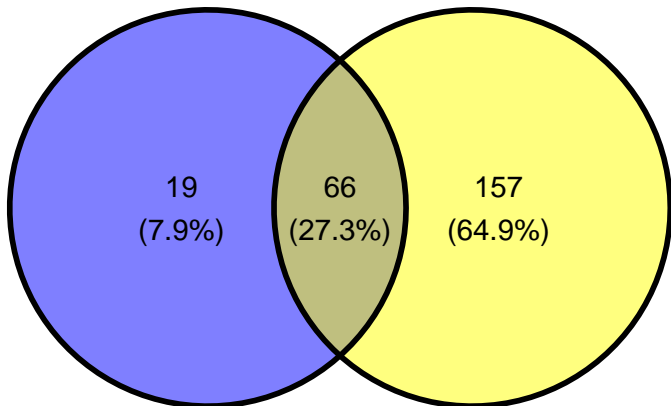

Supplement: Data S1 [file peerj-12-17859-s007.zip › FigureS6/B2 mlnc-2.pdf]
