# Supplementary material for: Cuproptosis related ceRNA axis AC008083.2/miR-142-3p promotes the malignant progression of nasopharyngeal carcinoma through STRN3
Source: PeerJ. 2024 Aug 12;12:e17859. doi: 10.7717/peerj.17859 (PMC11326429; doi:10.7717/peerj.17859)
Supplement: Data S1 [file peerj-12-17859-s007.zip › Figure7/A lncRNA-miRNA.pdf]

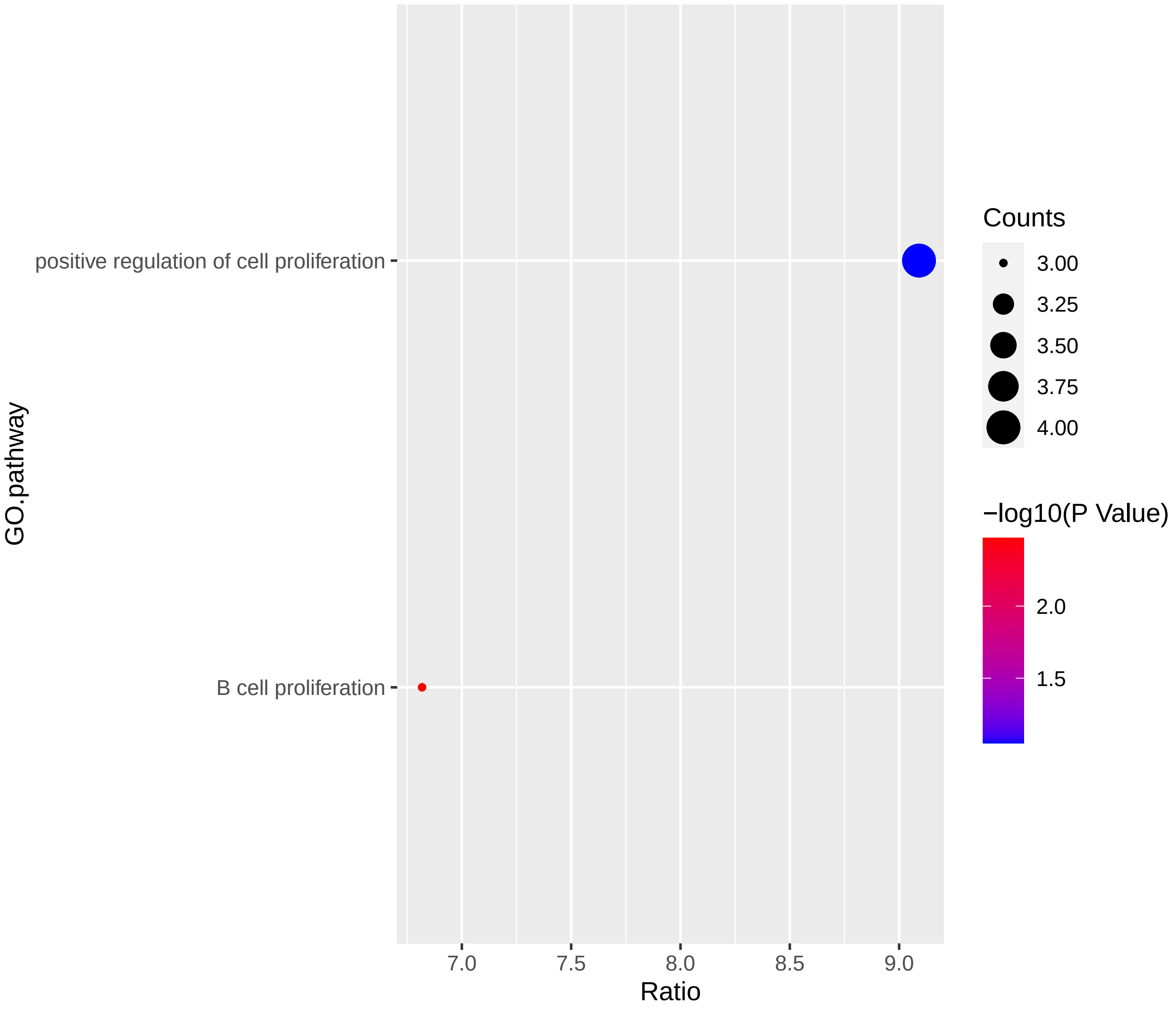

positive regulation of cell proliferation
B cell proliferation
GO.pathway
7.0
7.5
8.0
8.5
9.0
Ratio
Counts
3.00
3.25
3.50
3.75
4.00
−log10(P Value)
2.0
1.5
